# Nitrogen Stimulates the Growth of Subsurface Basalt-associated Microorganisms at the Western Flank of the Mid-Atlantic Ridge

**DOI:** 10.3389/fmicb.2016.00633

**Published:** 2016-05-03

**Authors:** Xinxu Zhang, Jing Fang, Wolfgang Bach, Katrina J. Edwards, Beth N. Orcutt, Fengping Wang

**Affiliations:** ^1^State Key Laboratory of Microbial Metabolism, School of Life Sciences and Biotechnology, Shanghai Jiao Tong UniversityShanghai, China; ^2^State Key Laboratory of Ocean Engineering, School of Naval Architecture, Ocean and Civil Engineering, Shanghai Jiao Tong UniversityShanghai, China; ^3^MARUM Center for Marine Environmental Sciences and Department of Geosciences, University of BremenBremen, Germany; ^4^Department of Biological Sciences, University of Southern CaliforniaLos Angeles, CA, USA; ^5^Bigelow Laboratory for Ocean SciencesEast Boothbay, ME, USA

**Keywords:** deep biosphere, geomicrobiology, iron oxidation, nitrogen stimulation, oceanic crust

## Abstract

Oceanic crust constitutes the largest aquifer system on Earth, and microbial activity in this environment has been inferred from various geochemical analyses. However, empirical documentation of microbial activity from subsurface basalts is still lacking, particularly in the cool (<25°C) regions of the crust, where are assumed to harbor active iron-oxidizing microbial communities. To test this hypothesis, we report the enrichment and isolation of crust-associated microorganisms from North Pond, a site of relatively young and cold basaltic basement on the western flank of the Mid-Atlantic Ridge that was sampled during Expedition 336 of the Integrated Ocean Drilling Program. Enrichment experiments with different carbon (bicarbonate, acetate, methane) and nitrogen (nitrate and ammonium) sources revealed significant cell growth (one magnitude higher cell abundance), higher intracellular DNA content, and increased Fe^3+^/ΣFe ratios only when nitrogen substrates were added. Furthermore, a *Marinobacter* strain with neutrophilic iron-oxidizing capabilities was isolated from the basalt. This work reveals that basalt-associated microorganisms at North Pond had the potential for activity and that microbial growth could be stimulated by *in vitro* nitrogen addition. Furthermore, iron oxidation is supported as an important process for microbial communities in subsurface basalts from young and cool ridge flank basement.

## Introduction

The largest hydrologically active aquifer on Earth is found in the top ~500 m of the fractured and permeable basaltic ocean crust (Fisher, 2005). However, little is known about the distribution, function and activity of microbial communities inhabiting this subsurface realm, which is among the least studied and most poorly understood of Earth’s major biological habitats (Schrenk et al., 2010). Microbial transformation of carbon, nitrogen and bioavailable metals (iron, manganese) may have significant impact on these global elemental budgets, and oxidation of reduced elements in the basalt would alter the ocean floor landscape over geological timescales (Bach and Edwards, 2003; Edwards et al., 2012).

To date, most investigations of subsurface ocean crust microbiology have taken place at the Juan de Fuca Ridge flank, which is characterized by relatively high temperatures (~65°C) and chemically reduced fluids derived from hydrothermal exchange between circulating seawater and basaltic basement (Cowen et al., 2003; Wheat et al., 2010; Lin et al., 2012). Functional gene analysis, carbon and sulfur isotopic signatures and laboratory incubations provide direct evidence that methane- and sulfur-cycling microbes are present in this environment (Lever et al., 2013; Robador et al., 2015), although the functions of other microbial community members of this chemical reduced environment are less clear (Orcutt et al., 2011; Jungbluth et al., 2013, 2014). Microbial diversity studies from a similar crustal environment on the Costa Rica Rift flank (similar in terms of crustal age—3.5 Ma at Juan de Fuca versus 6.4 Ma at Costa Rica Rift—and basement temperature—64°C versus 58°C) reveal a dominance of *Thiomicrospira* in the borehole observatory (Nigro et al., 2012). Alteration textures, N/C ratios and δ^13^C values indicate microbial activity in altered basaltic glass collected from this environment (Torsvik et al., 1998). By comparison to warm and reducing crustal environments, seafloor basaltic rocks exposed near the East Pacific Rise, the Loihi Seamount, and the Arctic mid-ocean ridge spreading center have revealed diverse bacterial communities (Lysnes et al., 2004; Einen et al., 2008; Santelli et al., 2008, 2009; Edwards et al., 2011; Jacobson Meyers et al., 2014). Furthermore, enrichment experiments with seafloor basalts under oxic conditions have revealed that *Marinobacter* spp. often are associated with iron oxidation (Edwards et al., 2003, 2004), and a monophyletic *Marinobacter* clade is suggested to represent iron-oxidizing facultative chemoautotrophs based on the phylogenetic analysis of the 16S rRNA gene (Kaye et al., 2011). These studies suggest that chemically reduced basalts are altered by bottom seawater and release geochemical energy, which supports microbial abundances on the rock surfaces that are 3 – 4 orders of magnitude higher than those in the overlying deep-sea water.

Previous microbial studies of oceanic crust have been limited to high temperature ridge flank or seafloor-exposed basaltic crust environments as introduced above (Fisk et al., 1998; Cowen et al., 2003; Lysnes et al., 2004; Santelli et al., 2008; Edwards et al., 2011), or to sites with mantle-type rock (Brazelton et al., 2010; Mason et al., 2010). Sediment-buried, subsurface basaltic crust in low-temperature ridge flank systems, which represents a more common hydrologically active type of ocean crust (Edwards et al., 2012), have not yet been studied for microbial community characterization. This type of environment was the focus of the recent Integrated Ocean Drilling Program (IODP) Expedition 336, which cored basaltic basement at “North Pond,” a site on the western flank of the Mid-Atlantic Ridge (Expedition 336 Scientists, 2012b; Orcutt et al., 2013; Supplementary Figure S1). This area was previously studied by ocean drilling (Becker et al., 2001) and site survey investigations (Langseth et al., 1992; Picard and Ferdelman, 2011; Ziebis et al., 2012) and revealed vigorous seawater circulation within young basaltic crust under a <300 m thick sediment pile (Expedition 336 Scientists, 2012b; Supplementary Figure S2a). Recent results indicated that ~210 μM of oxygen reacts in the upper basement at North Pond (Orcutt et al., 2013; Meyer et al., 2016), which had been suggested (Ziebis et al., 2012) and assumed (Bach and Edwards, 2003) previously; this consumption could be due to microbial activity. The geochemical composition of the subsurface crustal fluids in this area was found similar to that in the bottom seawater (Meyer et al., 2016). North Pond was thus targeted as an ideal place for studying crustal microorganisms, because of its young crustal age (8 million-year old), the high intensity of fluid flow, and relatively low temperatures (5 – 25°C) (Bach and Edwards, 2003; Edwards et al., 2012; Expedition 336 Scientists, 2012b).

To determine whether microbial life exists and is potentially active in the subsurface basement at North Pond, we set up a series of incubations of different basalt and sediment samples (**Table 1**) with variable addition of carbon and nitrogen substrates (**Table 2**) for enriching and isolating microorganisms. These enrichments allowed us to test the hypotheses that reduced basaltic rock coupled with oxidants from the seawater medium could provide sufficient energy for microbial communities to survive and proliferate (Lever et al., 2013), and that iron oxidation is an important energy producing process in the crust (Bach and Edwards, 2003). They also facilitated an assessment of potential nutrient limiting factors on microbial growth in subsurface basalt. Sterile controls and other measures of possible contamination were included to rule out artifacts of sample handling on the results. Phylogenetic analysis of the basalt bacterial communities coupled with isolation of neutrophilic iron-oxidizing bacteria documented the viability of a deep subseafloor biosphere hosted in young and cool oceanic crust.

**Table 1:** Characteristics of IODP Expedition 336 samples used in this study.

Sample	Hole	Depth (mbsf)	Description
2R-2E	U1383C	72.15	Sparsely plagioclase phyric pillow basalt, highly altered, 2 mm thick vein with red alteration
10R-1B	U1383C	144.81	Sparsely plagioclase-olivine phyric fine-grained basalt
30R-1A	U1383C	304.05	Aphyric, cryptocrystalline basalt
3R-4B	U1382A	117.82	Aphyric fine-to-medium-grained basalt
1H-2	U1382B	1.65	Nannofossil ooze from oxic section
6H-8	U1382B	49.88	Nannofossil ooze from anoxic section

**Table 2:** Concentrations of carbon and nitrogen substrate amendments in the initial sterile-filtered seawater medium.

Substrates amended	Final concentration
NH_4_Cl	1.5 mM
NaNO_3_	3.0 mM
NaHCO_3_	1.5 mM
CH_3_COONa	1.5 mM
CH_3_OH	2% [v/v]
CH_4_	20% [v/v] in headspace

## Materials and Methods

### Sample Collection, Contamination Assessment and Incubation Experiments

Basaltic basement and sediment samples were collected from North Pond during IODP Expedition 336 (**Table 1**; Supplementary Figures S1 and S2A). The methods for collection and processing of the samples, including quality and contamination assessment, are described elsewhere (Expedition 336 Scientists, 2012a) and detailed in the Supplementary Material. Briefly, to assess for the possibility of contamination from drilling, samples were checked for the presence of fluorescent microspheres used during coring, and a sample of the drilling mud used during drilling was collected for comparative microbial diversity analysis. Three basalt samples from Hole U1383C, one basalt sample from Hole U1382A, and two sediment samples from Hole U1382B which showed no evidence of microsphere contamination were used in this study (**Table 1**). Properties of these samples are described in more detail elsewhere (Expedition 336 Scientists, 2012c,d).

For the basalt enrichments, whole-round rocks were broken into smaller pieces shipboard using an ethanol- and flame-sterilized hydraulic press. Only interior pieces of rock samples were selected for microbiological study to avoid potential drilling mud contamination, as recommended elsewhere (Expedition 330 Scientists, 2012; Lever et al., 2013). Basalts were further ground into sand-sized fractions with a flame-sterilized steel percussion mortar, and transferred to sterile 18 mm × 150 mm glass tubes. In general, 2 cm × 2 cm × 2 cm of each rock sample was mixed with 5 mL of 0.22 μm-mesh filtered seawater collected on site at a water depth around 100 m. A carbon substrate (sodium bicarbonate, sodium acetate or methane) and/or a nitrogen substrate (ammonium chloride or sodium nitrate) were added at a final concentration of 1.5–3 mM or 20% [vol/vol] headspace for enrichments (**Table 2**); one control treatment without carbon or nitrogen addition was also included. All tubes were capped with butyl rubber stoppers with filter-sterilized air in the headspace. All enrichments were incubated at 10°C in the dark. For the sediment enrichments, similar procedures were applied as described in Supplementary Material. After 6 months of incubation, 2 mL of thoroughly mixed slurry which contained suspended rock particles/sediment and seawater was transferred and preserved in an equal volume of 1 × phosphate buffered saline (PBS)/ethanol at -20°C until analysis. Subsamples of the enrichments at the end of the incubations were also preserved frozen at -20°C for DNA analysis (subsamples of the original material were also preserved frozen for comparison).

A parallel series of incubations were conducted with a double autoclaved basalt sample from 336-U1382A-3R-4B as a sterile control. The crustal materials recovered from Hole U1383C and U1382A were similar in composition (Expedition 336 Scientists, 2012b), and the crust samples recovered were very limited, therefore only one sterile control was set in this study. No amplification of bacterial/archaeal 16S rRNA genes from DNA extracts, nor detection of microbial cells by epi-fluorescence microscopy from the filtered seawater medium with double autoclaved 3R-4B enrichments (methods described in more detail below), indicated that 0.22-μm mesh was appropriate for sterile seawater media in this study, similar to previous reports (Meron et al., 2011; Zeng and Chisholm, 2012; Reveillaud et al., 2014).

### Concentrations of Dissolved Ions

Fe^2+^ and total iron (ΣFe) from the original 30R-1A and 3R-4B samples as well as the enrichments were measured by spectrophotometer (DR5000, Hach, USA) using Ferrous Iron Reagent Powder Pillows and FerroZine Iron Reagent Solution Pillows according to manufacturer instructions, respectively. Briefly, 40 – 60 μL (according to desired concentration) of thoroughly mixed slurry was added with an equal volume of 6 M anaerobic hydrochloric acid to extract solid phase and absorbed iron (Roden and Zachara, 1996). This mixture was left in the dark for 24 h. The sample was then filtered (0.45-μm mesh Pall filter) and diluted up to 25 mL with anaerobic double-distilled water. The reagent powder was added to the tube and inverted to mix. All procedures were performed in an N_2_-sparged anaerobic box (Coy) to avoid Fe^2+^ oxidation. After reaction for 3 min, a sample cell was filled with 10 mL sample to measure absorbance using program 255 for Fe^2+^ and program 260 for total iron according to manufacturer instructions (Hach, USA). Ammonium, nitrate, nitrite, iron and hydrogen ion (i.e., pH) concentrations were measured at the end of the enrichment incubations by allowing the slurries to settle, filtering the overlying supernatant through a 0.22-μm mesh GTBP membrane (Millipore, USA), and freezing the filtrate at -20°C until analysis. Ammonium was measured by the Berthelot color reaction method as described by Weatherburn (1967) using a spectrophotometer (DR5000, Hach, USA). The detection limit of this method is 2.7 μM. Nitrate and nitrite were determined by ion chromatography using a Metrohm MIC-1 Advanced IC System with an 819 IC Detector (Metrohm, Switzerland). Before measurement, each stored sample was diluted 10 times with double-distilled water and passed through an Ag column (ANPEL, China) to eliminate high Cl ion contents. A Metrosep A Supp 5-250 column (Metrohm) was used with a solution of 3.2 mM Na_2_CO_3_ and 1.0 mM NaHCO_3_ as eluent. The detection limits of this method for nitrate and nitrite are 1.6 and 0.1 μM, respectively. The pH of the medium was measured using a S220 SevenCompact^TM^ pH meter (Mettler-Toledo, Switzerland) according to manufacturer instructions.

### Cell Enumeration and Fluorescence Intensity Measurement

Cell enumeration was performed after a cell extraction procedure applied for low-biomass samples following a method modified from Kallmeyer et al. (2008). Since the density of basalt is high (2.43 g cm^-3^) and microbial cells may not evenly distributed in the whole-round rock, the collected rocks were crushed into smaller pieces and interior pieces were further ground into sand-sized fractions and pooled according to the initial sample ID. Each reagent used before the cell enumeration steps was filter sterilized through a 0.22 μm-mesh membrane filter (Millipore, USA). The cell enumeration blank was performed without a sample and processed with the same steps as the basalt samples. Cells were counted at 1000× magnification using an epi-fluorescence microscope (Nikon, ECLIPSE 90i, Japan) with a blue filter set. Each sample was extracted and counted in triplicate. An average of 135 fields of view was counted for each membrane. The area of each field of view was set at 10,000 μm^2^, and the detection limit was ~10^4^ cells cm^-3^ for a 95% probability of detecting at least 1 cell as described by Kallmeyer et al. (2008). In addition, quantitative PCR analysis of the bacterial 16S rRNA gene was performed on DNA extracts from the 10R-1B samples as an additional measure of cell abundance. Details of cell extraction and enumeration are provided in Supplementary Material. For the measure of cellular fluorescence intensity, the same cell extraction, filtration and staining procedure were applied as described above. To avoid dye fading, each surveyed area on the membrane was exposed to excitation light <5 s, and an area that was distant from the previous exposed area was chosen. Images were taken and analyzed by NIS-Elements AR software (Nikon, Japan), following the same parameters for all samples.

### Statistical Analysis of Iron Concentrations and Fluorescence Intensity

Statistical significance between samples were analyzed using Mann–Whitney *U*-test (SPSS 13.0). Differences were considered significant when *P* < 0.05.

### Nucleic Acid Extraction, Amplification, and Sequencing of 16S rRNA Gene

Basalt genomic DNA extraction, amplification of bacterial/archaeal 16S rRNA gene V4 region and high-throughput sequencing of PCR amplicons were detailed in Supplementary Material. The 16S rRNA gene amplicons containing unique 8-mer barcodes used for each sample were pooled with equal concentration, and sequenced on an Illumina MiSeq platform using 2 × 250 bp cycles and MiSeq Reagent Kit v2 (500 cycle, Illumina, USA) according to manufacturer instructions. All procedures were performed under a laminar flow hood, and parallel blank extractions without sample added were carried out to identify possible contamination during extraction and from extraction kits; all extraction blanks and procedure negative controls were free of contamination. PCR primers are listed in Supplementary Table S1.

### 16S rRNA Gene Analysis

Sequences were aligned with PyNAST (Caporaso et al., 2010a; Version 1.2.2) and clustered into operational taxonomic units (OTUs) at 97% sequence similarity cutoff using usearch61 with default parameters in the QIIME software pipeline (Caporaso et al., 2010b; Version 1.9.0). OTUs were assigned to genus level using the Greengenes database (DeSantis et al., 2006; Version gg_13_5). Chimeras were detected with the UCHIME program (Edgar et al., 2011; Version 4.2) using default parameters and removed from further analysis (the percentage of chimeric sequences were between 6.2 and 9.2%). Cluster analysis of the microbial community structure was performed in *R* based on Bray–Curtis matrix by using average linkage (R Core Team, 2015; Version 3.2.0). Phylogenetic trees were constructed in QIIME using the FastTree method, and the Shimodaira–Hasegawa test was used to estimate the reliability of each branch with 1000 resamples (Price et al., 2010; Version 2.1.3). Sequences covering the V4 region of 16S rRNA gene from crustal environments of Juan de Fuca Ridge flank (Jungbluth et al., 2013, 2014), Costa Rica Rift flank (Nigro et al., 2012), East Pacific Rise (Santelli et al., 2008, 2009), Atlantis Massif (Mason et al., 2010) and Loihi Seamount (Edwards et al., 2011) with similarity to the North Pond sequences were included in the trees. All sequence data have been deposited in the National Center for Biotechnology Information Sequence Read Archive under the accession number SRP063586.

To assess potential contaminating sequences from reagent/kit, a low-biomass contaminant database was constructed using sequences from Tanner et al. (1998), Kulakov et al. (2002) and Barton et al. (2006). All of the OTUs that were assigned to the same taxa with the contaminating sequences were used to construct phylogenetic trees using the same method as described above. A representative set of sequences for each OTU were used due to the high sequence number. If an OTU was closely related to sequences from the low-biomass contaminant database, it was further compared with the contaminating sequence using ClustalW Alignment (Thompson et al., 1994; Version ClustalW2) to give a sequence similarity value. As suggested by Barton et al. (2006), any sequences that demonstrated >98% sequence similarities to the contaminant database were flagged as possible contaminants. Detailed assessment results are provided in Supplementary Material.

### Isolation, Identification and Growth Substrate Test of *Marinobacter* sp. NP-6

The gradient tube method (Emerson and Floyd, 2005) was used to culture iron-oxidizing bacteria. The top layer was a semisolid mineral media containing artificial seawater (ASW, pH 7.0) and 0.15% wt/vol low-melting agarose. The bottom layer was a mixture of equal volume of ASW and a reduced iron substrate that was solidified by adding 1% wt/vol regular agarose. This created a diffusion gradient of oxygen from the top and reduced iron from the bottom. The tubes were incubated at 15°C in the dark. The initial gradient tubes were inoculated with slurry from a 30R-1A enrichment (ammonium amendment) and subsequent dilution-to-extension transfers were performed until pure cultures were obtained. Growth of the isolate was then tested on reduced iron substrates including ferrous sulfide [FeS, solid, synthesized as described by Emerson and Floyd (2005)], ferrous carbonate [FeCO_3_, solid, synthesized as described by Hallbeck et al. (1993)], basalt (0.05 g powder per tube, double-autoclaved samples from Hole U1383C), and zero-valent iron (0.01 g powder per tube, purchased from a commercial vendor) by the gradient tube method. The respiratory inhibitor sodium azide was amended to a final concentration of 1 mM in gradient tubes. Anaerobic media were prepared by sparging N_2_ gas for 30 min in serum bottles before autoclaving. Scanning electron microscopic (SEM) images were taken with a JEOL JSM-7001 field emission SEM after dehydration and critical point drying procedures as described elsewhere (Orcutt et al., 2011). Genomic DNA of this strain was extracted using a FastDNA^TM^ SPIN Kit for Soil (MP Biomedicals, Santa Ana, CA, USA) according to the manufacturer’s instructions, and amplification and sequencing of near full-length 16S rRNA genes were carried out as described elsewhere (Wang et al., 2014). The sequence was analyzed using the Basic Local Alignment Search Tool (Altschul et al., 1990) against the NCBI database. The 16S rRNA gene sequence of this strain was deposited in the GenBank sequence database under the accession number KJ914666.

## Results

### Growth Tests with Different Substrates

**FIGURE 1:**
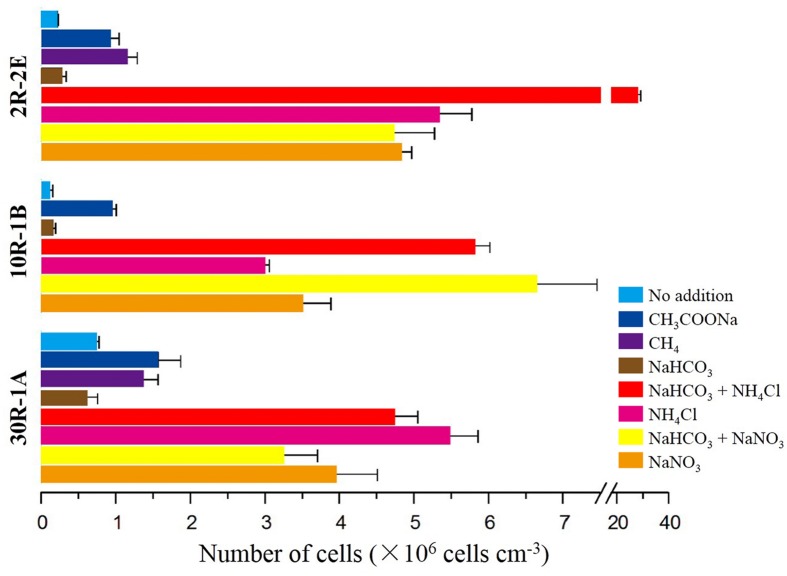
**Cell abundance on subsurface basalts after 6 months of incubation with variable carbon and nitrogen substrates.** Three sections of basaltic rock from Core U1383C are incubated and counted, 2R-2E (72.15 mbsf), 10R-1B (144.81 mbsf), and 30R-1A (304.05 mbsf). Color bars represent combinations of different carbon and/or nitrogen substrates added as shown on the left. The “No addition” treatments are negative control incubations without any added substrate.

To test whether microbial cells have the potential for growth in subsurface basalts, a series of incubation experiments with different carbon and nitrogen substrates were performed. In general, higher cell concentrations occurred in enrichments after 6 months with the addition of either nitrogen only or with a combination of sodium bicarbonate and a nitrogen substrate (**Figure 1**). For the topmost sample 2R-2E, the cell concentrations in nitrogen enrichments were between 4.71 × 10^6^ and 2.78 × 10^7^ cells cm^-3^ (Supplementary Table S2). In contrast, enrichments with sodium bicarbonate, sodium acetate or methane addition without nitrogen gave lower cell concentrations between 2.54 × 10^5^ and 1.13 × 10^6^ cells cm^-3^. The highest cell concentrations occurred in enrichments with both sodium bicarbonate and ammonium chloride addition. Results for samples 10R-1B and 30R-1A from the middle and bottom sections of Hole U1383C yielded similar trends related to enrichments with nitrogen addition having higher cell numbers, although there were differences in which nitrogen compound lead to the most growth (**Figure 1**). For 10R-1B, incubations with the addition of sodium bicarbonate with either ammonium chloride or sodium nitrate showed the highest cell concentrations (roughly 6 × 10^6^ cells cm^-3^, values not statistically different, Supplementary Table S2), while the incubations without nitrogen addition were within the range of 9.76 × 10^4^ – 9.37 × 10^5^ cells cm^-3^. For 30R-1A, highest cell concentrations were found in incubations with ammonium chloride addition, while the lowest cell concentrations were enrichments with sodium bicarbonate addition. These trends of higher abundance in nitrogen-amended samples were supported by the observation of higher bacterial 16S rRNA gene copy numbers in such samples as compared to non-nitrogen-amended samples, as determined by quantitative PCR (Supplementary Figure S3).

In general, microbial cell concentrations in basalt incubations with the addition of a nitrogen substrate were roughly 1 – 2 orders of magnitude higher than in those that had only carbon added (**Figure 1**). This trend could be observed in samples from all subseafloor depths. Cell concentrations in incubations with ammonium chloride addition were higher than where sodium nitrate was added in samples 2R-2E and 30R-1A. In contrast, sample 10R-1B showed the opposite trend. Incubations with the addition of either acetate or methane only gave cell counts that were slightly higher but still of the same order of magnitude as in incubations without any substrate addition, and those incubated with only additional sodium bicarbonate, suggesting that these carbon sources did not stimulate cell growth alone. Similar enrichments with two sediments from nearby Hole U1382B (1H-2 and 6H-8; **Table 1**) revealed no stimulation of cell growth in sediment amended with methane, methanol or nitrogen, although slightly higher cell concentrations (~30% higher) were observed when amended with acetate or bicarbonate with nitrogen amendment (Supplementary Figure S4). The overall cell concentrations per volume were higher in the sediment enrichments than in the rock enrichments.

### Geochemical Substrates Utilization in Enrichments

To assess the utilization of the geochemical substrates in the enrichments that might reflect microbial activity, we measured concentrations of dissolved and total iron, nitrate, nitrite, ammonium, and pH. Increased Fe^3+^/ΣFe ratios were observed in enrichments with nitrogen substrate addition after 6 months (Supplementary Figure S5). In sample 10R-1B, the significantly highest Fe^3+^/ΣFe ratio (0.81) occurred in the ammonium addition incubation. This ratio was 3.2 and 6.9% higher (*P* < 0.01) than the no addition treatment and in the initial rock, respectively. In sample 30R-1A, the highest ratio (0.82) was in the nitrate addition, which was 7.3 and 11.8% higher than the no addition treatment and in the initial rock, respectively (*P* < 0.01). In 2R-2E, the highest Fe^3+^/ΣFe ratio occurred in the bicarbonate only addition incubation, but nitrogen addition incubations still had higher ratios than in the no addition treatment and in the rock prior to incubation. A parallel double autoclaved enrichment from sample 3R-4B was also measured for Fe^3+^/ΣFe ratios to test the extent of abiotic iron oxidation. A 10% increase of Fe^3+^/ΣFe ratios were observed after incubation compared to the initial rock. However, enrichments with varied carbon and/or nitrogen substrates did not show any statistically significant trend as was observed in the live incubations, except that the nitrate-amended enrichment resulted in a slightly higher Fe^3+^/ΣFe ratio (0.79). The pH of the seawater medium was 7.8 – 8.0 after incubation for 6 months as compared to 7.5 before incubation (data not shown), so pH changes should have only have minimal effect on iron solubility.

Nitrate, nitrite and ammonium concentrations were measured after 6 months of incubation (Supplementary Table S3). In the enrichments with 1.5 mM ammonium amendment, approximately 28 – 50% of ammonium was consumed. A slight accumulation of ammonium (up to 0.1 mM) was observed from nitrate amendment enrichments in sample 2R-2E. Rocks incubated with carbon substrates did not show increased ammonium concentrations in nitrate enrichments, which gave similar values as the no addition incubations and the sterile control. The percentage of nitrate consumed was lower in nitrate amendment enrichments (9 – 36% decrease, Supplementary Table S3), although the total decrease was of the same order of magnitude considering that twice the amount of nitrate was added (3 mM) as compared to ammonium (1.5 mM). No accumulation of nitrate was observed from samples with ammonium amendment. Acetate enrichments from all of the three samples showed slightly higher nitrate (>40 μM) than in the no addition controls. Nitrite was only detected in low concentrations (2 – 3 μM) in enrichments from 10R-1B. Concentrations of ammonium and nitrate in the bicarbonate enrichments and no substrate addition controls were in the same range with the sterile samples from 3R-4B.

### Cell Morphology

**FIGURE 2:**
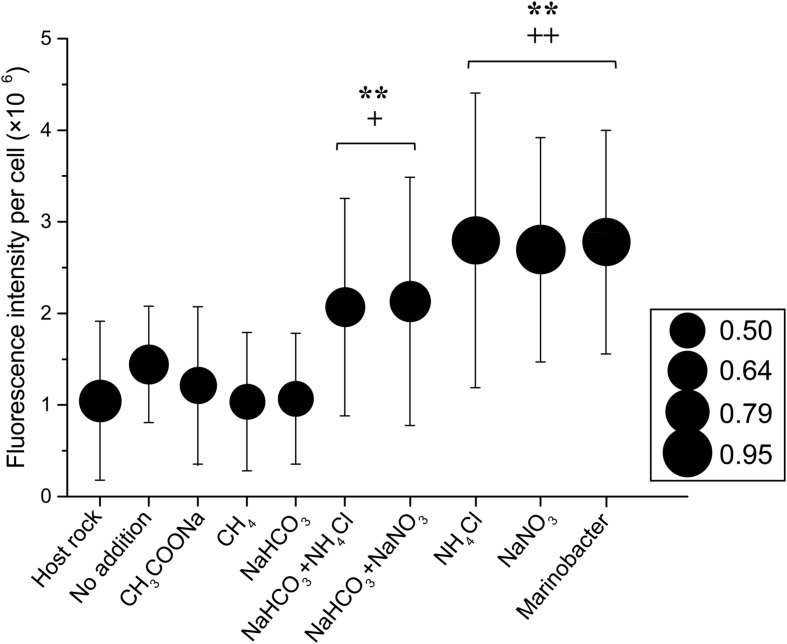
**Cellular fluorescence intensity and cell size as a function of enrichment substrate on a subset of basalt enrichments (sample 30R-1A).** The circle size indicates the average cellular area (mm^2^) as measured by epi-fluorescence microscopy. The standard deviation for the cellular area of each sample is roughly 46% based on the following number of cells counts for each treatment: Host Rock (*n* = 28); No addition (*n* = 41); CH_3_COONa (*n* = 38); CH_4_ (*n* = 62); NaHCO_3_ (*n* = 43); NaHCO_3_ + NH_4_Cl (*n* = 39); NaHCO_3_ + NaNO_3_ (*n* = 36); NH_4_Cl (*n* = 35); NaNO_3_ (*n* = 38). Symbols ++ and + indicate analysis of variance *P*-values of <0.01 and <0.05, respectively, versus Host rock or No addition; and ^∗∗^ indicate *P* < 0.01 versus CH_3_COONa, CH_4_ or NaHCO_3_.

In all samples, most of the cells identified by epi-fluorescence and scanning electron microscopy were rod-shaped or spherical (data not shown), with a typical diameter of 0.5 μm and ranging from 0.3 to 1.0 μm (**Figure 2**). Some cells were attached to rock particles while most cells were unattached, likely due to the method of preparation (Kallmeyer et al., 2008). The fluorescence intensity of cells stained with SYBR Green I dye from nitrogen substrate additions was significantly higher than those without nitrogen addition, irrespective of average cell size (**Figure 2**). In sample 30R-1A, highest fluorescence intensity and cellular area increases occurred in incubations with only ammonium chloride or sodium nitrate addition. The cellular fluorescence intensity in those treatments was similar to those observed in a *Marinobacter* strain isolated from sample 30R-1A (described in more detail below). Cells from the original samples and without substrate addition had similar and lower fluorescence intensity values, and cells from the carbon amendment had the lowest fluorescence intensity and cellular area (**Figure 2**).

### Bacterial Diversity

Between 8,568 and 161,890 partial bacterial 16S rRNA gene sequences were obtained from the 30R-1A samples after sequence quality filtering and chimera removal (Supplementary Figure S6). No archaeal 16S rRNA gene sequences were obtained although different archaeal primers were tested (see Supplementary Table S1), suggesting a low abundance of archaea in the sample. Across all 30R-1A samples, the most prevalent sequence group was related to *Marinobacter* of the Gammaproteobacteria (Supplementary Figure S6). The *Marinobacter*-related sequences were abundant and diverse, containing 254 different OTUs (97% sequence similarity cutoff). *Sediminibacterium* of the Bacteriodetes phylum was not as prevalent in the enrichment samples as those in the original sample. *Alteromonas* (Gammaproteobacteria) and Flammeoviraceae (Bacteroidetes) related sequences increased in relative abundance in the carbon-amended enrichments. Other groups were relatively constant in low abundance across treatments. Cluster analysis of the sequence data indicated that incubations with the addition of a nitrogen substrate formed one group, and those with only carbon substrate added grouped together (Supplementary Figure S6). The nitrogen addition incubations formed two separate subclusters separated by nitrate or ammonium addition.

**FIGURE 3:**
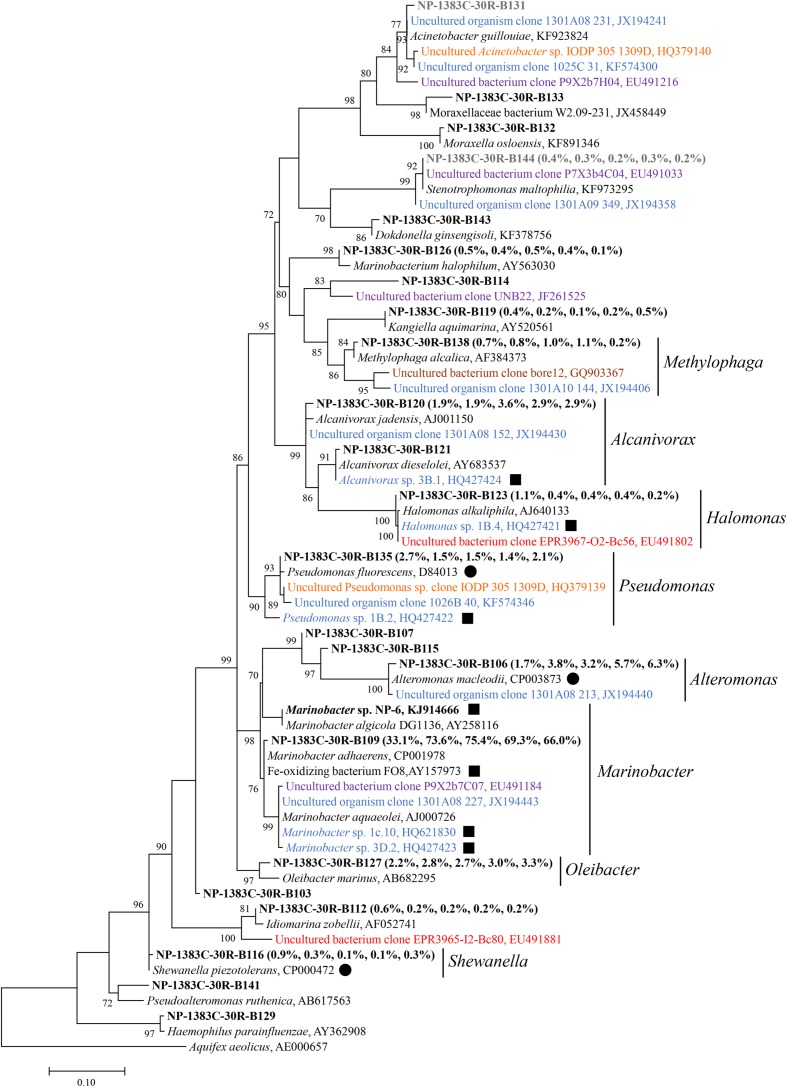
**Phylogenetic tree of Gammaproteobacteria related 16S rRNA gene sequences from host rock sample 30R-1A in comparison to sequences from cultivated species and other environmental studies.** The sequence data of 30R-1A host rock are retrieved from Zhang et al. (2016), and are highlighted in bold font. The numbers in parentheses indicate percent abundance of the reads clustered in genera followed by the order “Host rock” “NaHCO_3_ + NH_4_Cl” “NH_4_Cl” “NaHCO_3_ + NaNO_3_” “NaNO_3,_” respectively. A representative sequence from sample 30R-1A for each genus is shown due to the high sequence number. Sequences from isolates or other environmental studies identified by Genbank accession number. Filled squares indicate known iron-oxidizing bacteria, and filled circles indicate known iron-reducing bacteria. Sequences retrieved from Atlantis Massif are in orange, East Pacific Rise in red, Loihi Seamount in purple, Juan de Fuca Ridge flank in blue, crustal fluids of Costa Rica Rift flank in brown. Potential kit contaminating sequences are in gray. The number at each branch indicates local support value, with only values >70 shown. The 16S rRNA gene of *Aquifex aeolicus* (AE000657) is used as outgroup. The scale bar indicates 0.1 nucleotide substitutions per site.

Phylogenetic analysis revealed that Alpha-, Beta-, and Gamma-Proteobacteria account for the majority of the bacterial diversity, each of them being distributed among more than 13 different families (**Figure 3**, Supplementary Figures S7 and S8). Sequences from North Pond were closely related to known iron-oxidizing or iron-reducing bacteria. For instance, *Marinobacter*-related sequences from the basaltic samples clustered with known iron oxidizing bacteria (**Figure 3**). Few sequences retrieved from previously studied crustal environments (Santelli et al., 2008, 2009; Mason et al., 2010; Edwards et al., 2011; Nigro et al., 2012; Jungbluth et al., 2013, 2014; Robador et al., 2015) were related to sequences from the North Pond basalts, and these did not group with the most abundant OTUs.

### Isolation and Characterization of Iron-oxidizing Bacteria

Gradient tube method was used to isolate iron-oxidizing bacteria under microaerophilic, neutrophilic conditions (Emerson and Floyd, 2005). Notably, one pure culture was isolated from basalt sample 30R-1A (designated isolate strain NP-6), belonging to the genus *Marinobacter*. The 16S rRNA gene of this strain grouped within the second most abundant *Marinobacter* OTU from the 30R-1A basalts and had 100% sequence similarity with the OTU. This OTU clustered with a monophyletic *Marinobacter* clade suggested to represent iron-oxidizing facultative chemoautotrophs (Kaye et al., 2011) with greater than 99% sequence similarity to a representative sequence from this clade (Genbank accession number AY258116; **Figure 3**). *Marinobacter* are known as biogeochemical “opportunitrophs” that can utilize multiple carbon sources (Handley and Lloyd, 2013), and they are also known for their ability to oxidize iron under aerobic/anaerobic neutrophilic conditions (Edwards et al., 2003; Smith et al., 2011; Bonis and Gralnick, 2015).

**FIGURE 4:**
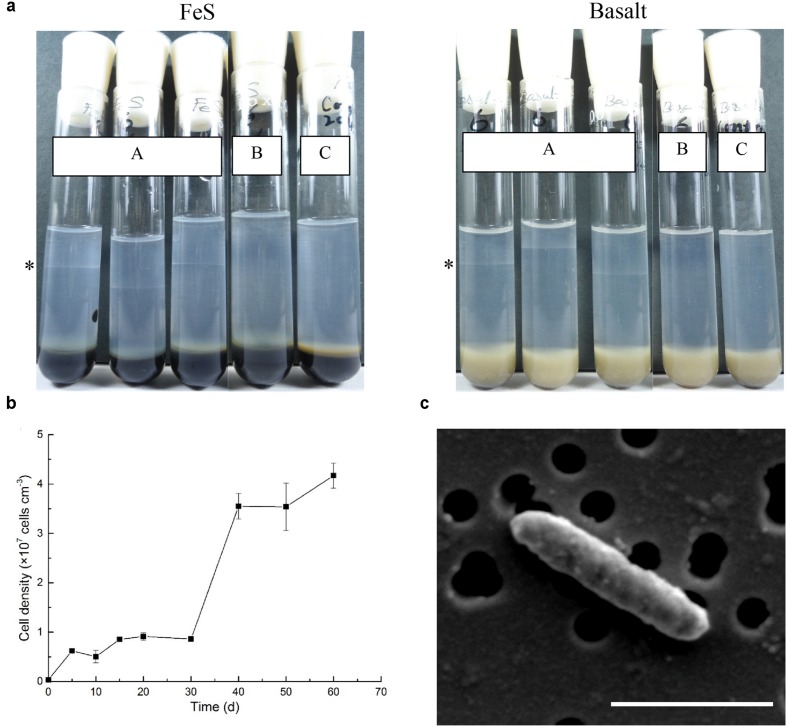
**Characterization of *Marinobacter* sp. NP-6 isolated from 30R-1A. (a)** Tubes (A) indicates cells were grown in gradient tubes with different iron sources (*n* = 3). The asterisk indicates position of the cell band. Tubes (B) indicates cells were grown with 1 mM sodium azide in gradient tubes. Tubes (C) indicates abiotic control. **(b)** Growth curve of this strain from FeS-based gradient tubes. The growth condition was 20°C, in dark. Three replicate tubes were performed for each point. **(c)** Scanning electron micrograph of this strain from FeS-based gradient tubes. The black circles indicate holes on a 0.22 μm membrane filter. Accelerating voltage, 5 kV. The scale bar indicates 1 μm.

The ability of the isolate strain NP-6 to oxidize iron was further tested by using different reduced iron substrates (ferrous sulfide, basalt, ferrous carbonate, and zero-valent iron). After 2 weeks incubation, this strain was observed to be capable of chemolithotrophic growth on all iron substrates at neutral pH by forming a band at the oxic-anoxic interface (**Figure 4a**). Maximum growth rate of strain NP-6 under the microaerobic iron-oxidizing condition occurred between 30 and 40 days, with a doubling time of 4.9 days at 20 °C (**Figure 4b**). Growth was not observed when using the respiratory inhibitor sodium azide in the gradient tubes, resulting in similar conditions as the abiotic control. Heterotrophic growth of this strain was observed in Marine Broth 2216 medium (contained complex organics, purchased from BD, USA) and in 5 mM acetate-amended ASW medium under aerobic condition, respectively (data not shown), indicating that this strain was a facultative chemoautotroph. However, anaerobic growth was not supported by Fe^2+^ (i.e., FeS, FeCO_3_, FeCl_2_) or acetate in ASW medium, and growth was not observed in Marine Broth 2216 medium, when using nitrate as the electron acceptor. Scanning electron microscopy showed strain NP-6 had rod shaped cells with a diameter of approximately 1.2 μm× 0.3 μm under iron-oxidizing conditions (**Figure 4c**), which had similar morphology to the cells reported for the enrichment samples. Biogenic mineral structures produced by some known iron oxidizers (i.e., stalks and sheaths) was not found in this strain.

## Discussion

### Microbial Growth Stimulated by Nitrogen Availability

The stimulation of cell growth in basalt-associated microbial communities by nitrogen addition observed in this study (**Figure 1**) contrasts with trends observed in the oligotrophic sediments used in this study (Supplementary Figure S4) and with those reported previously for organic rich subsurface sediments (Morono et al., 2011). In the previous study, carbon and nitrogen assimilation activities in individual microbial cells from deep subsurface sediments were examined, and it was suggested that microbial growth in deeply buried sediment is limited by energy rather than the availability of carbon/nitrogen compounds. Moreover, it was suggested that the sediment microorganisms preferentially require nitrogen assimilation for recovery *in vitro* in spite of sufficient ammonium availability (15 mM). While we have not estimated cellular nitrogen or carbon assimilation here, our study showed that nitrogen (nitrate or ammonium) stimulates growth of microorganisms in the basalt enrichments.

Nitrogen limitation in basalts could be expected, considering that basalts contain less than 0.01% nitrogen (Marty, 1995; Busigny et al., 2005), so the predominant source of nitrogen would be from the fluids circulating through the crust. Approximately 25 μM of total dissolved nitrogen (TDN) were detected in the subsurface crustal fluids of Hole U1383C (Meyer et al., 2016). The concentrations of readily available nitrogen sources in the fluids would likely decrease over time through consumption to levels that limit microbial growth, although bottom seawater TDN gave similar values as the crustal fluids (Meyer et al., 2016). Meanwhile, it would be difficult for basalt-hosted microorganisms to have access to the fluid-derived nitrogen sources, due to the low porosity of the basaltic crust (~4%) (Expedition 336 Scientists, 2012d; Zhang et al., 2016). A geochemical study by Torsvik et al. (1998) showed that the N/C ratios of altered basalts on the Costa Rica Rift flank were comparable to those of nitrogen-starved bacteria. In the nitrogen-amended enrichments in this study, concentrations of ammonium or nitrate were never less than 0.7 mM, indicating that substrate limitation was not an issue after 6 months of incubation. Finally, the detection of trace amount of nitrite for the 10R-1B samples (Supplementary Table S3), the observation of higher cell concentrations with nitrate amendment for 10R-1B as compared to higher cell concentrations with ammonium for samples 2R-2E and 30R-1A (**Figure 1**) suggest that the microbial communities in these samples have different strategies for growth.

It is not clear from our results whether ammonium was used by basalt-associated microbial communities for biosynthesis alone (for example, through amino acid biosynthesis), or also for energy production through nitrification. As compared to the sterile control, 28 – 50% of the ammonium added to the basalt samples was consumed after 6 months, but this did not correspond to an increase in nitrate or nitrite concentrations as would be expected if nitrification had occurred (Supplementary Table S3). The lack of observation of known nitrifying organisms, such as *Nitrosomonas*, *Nitrosospira*, or *Nitrococcus* in the 16S rRNA gene libraries (Supplementary Figure S6) suggests that nitrification as an energy-generating process was not occurring in the enrichments. However, if active denitrification was occurring in the enrichments in addition to nitrification, then any produced nitrate would have been converted to dinitrogen, which could also explain the lack of nitrate build up in the ammonium enrichments.

By comparison, nitrate was consumed to some degree in the nitrate-amended enrichments (Supplementary Table S3). Nitrate reduction could occur through either dissimilatory/assimilatory nitrate reduction to ammonia or denitrification to dinitrogen. Dissimilatory nitrate reduction to ammonia (DNRA) and denitrification are traditionally thought of as anaerobic processes, but recent studies demonstrated that both of these processes could also happen under aerobic conditions (Gao et al., 2009; Morley and Baggs, 2010). While oxygen concentrations were not measured in our experiments, it is possible that they remained oxic in bulk, although perhaps with partial anoxic niches. The observed slight accumulation of ammonium (up to 0.1 mM) in some of the nitrate-amended treatments (Supplementary Table S3) suggests that DNRA may have been occurring, but this could not explain all of the nitrate consumed. DNRA and denitrification abilities are commonly found in *Marinobacter*, *Alteromonas*, and *Pseudomonas* species (Knowles, 1982; Nakano et al., 2010; Handley and Lloyd, 2013), which were observed in our samples (Supplementary Figure S6). Combined with the observation of no nitrate build up in ammonium-amended treatments, it is likely that denitrification occurred in our samples. Assimilatory nitrate reduction to ammonia, which is further incorporated into cell material, may have been responsible for the remainder of the decrease in nitrate.

As compared to unamended controls, addition of acetate or methane at millimolar concentrations stimulated microbial abundance in incubations slightly, whereas addition of bicarbonate without nitrogen did not stimulate microbial growth (**Figure 1**). This indicates that carbon fixation from bicarbonate is not a dominant growth-inducing metabolic process in basalt-associated microbial communities. Although theoretical calculations suggested that chemolithotrophic reactions could support autotrophy in basalt environments (Bach and Edwards, 2003), these results indicated autotrophic processes were overshadowed by heterotrophic reactions. A recent study of the potential for carbon fixation in North Pond basalts documented very low to undetectable rates of carbon fixation in this environment (Orcutt et al., 2015). The increase in microbial abundance with acetate amendment suggests that basalt-associated biofilms support heterotrophic metabolisms. Heterotrophy was further supported by the growth of isolate strain NP-6 in organic rich media, and the observation of a net decrease in dissolved organic carbon in crustal fluids as compared to bottom seawater (Lin et al., 2012, 2014). It is possible that the filter-sterilized seawater used in the enrichments contained some dissolved organic carbon (although at a presumably low relative concentration, considering the location of North Pond under the North Atlantic Gyre) that may have stimulated growth, as the no addition enrichments had slightly higher cell concentrations compared to the original samples, although millimolar additions of carbon did not spur much more growth, suggesting that dissolved organic carbon was not limiting for cell growth.

### Iron Oxidation Supporting Microbial Communities in Young, Cool and Oxic Crust

Multiple lines of evidence suggest that iron oxidation is a dominant chemolithotrophic pathway supporting life in subsurface basalts from the cool and oxic basement environment at North Pond. First, microbial diversity analysis of sample 30R-1A revealed that bacteria within the *Marinobacter* dominated 16S rRNA gene sequence libraries, with relatively close sequence similarity to cultured iron-oxidizing bacteria (**Figure 3**; Supplementary Figure S6). While these bacterial groups are cosmopolitan in the ocean, they are also often detected and isolated from crustal samples in other studies (Edwards et al., 2003; Smith et al., 2011; Bonis and Gralnick, 2015; Hirayama et al., 2015). Second, an isolate capable of iron oxidation under chemolithotrophic, neutrophilic conditions was obtained from a subsurface basalt sample (**Figure 4**), and the 16S rRNA gene of this strain showed highest sequence similarity to *Marinobacter* abundant in the enrichments experiments and the original sample (**Figure 3**). Third, enrichments of basalts with nitrogen substrates, which had higher cell concentrations than non-nitrogen amended treatments (**Figure 1**), also had higher Fe^3+^/ΣFe ratios (Supplementary Figure S5), indicating iron oxidation supporting growth in these enrichments. While it may be tempting to extrapolate the potential rates of iron oxidation observed from the enrichments to *in situ* processes, this is complicated by the possible stimulation of biomass from the enrichment conditions (which included changed pressure, temperature, nutrients, etc.) and by the generation of fresh surface area as compared to *in situ* conditions. Microorganisms in the environment are normally attached to rock surfaces or distributed along veins or cracks (Fisk et al., 1998; Thorseth et al., 2003). In these experiments, microbial cells could easily have access to nutrients and energy released from core sections of crushed rocks, which facilitated higher rates of nutrient and energy fluxes.

Earlier work demonstrated an overall increase in iron oxidation state with age of oceanic crust, which was theorized to support lithotrophic growth under oxic or denitrifying conditions (Bach and Edwards, 2003). Others have documented that iron oxidation is a viable metabolic pathway for *Marinobacter* growing on basalt as an exclusive chemolithotrophic energy source (Edwards et al., 2003; Smith et al., 2011). Although abiotic iron oxidation cannot be ruled out in the enrichments [and indeed, a relatively high Fe^3+^/ΣFe ratio in the nitrate-amended sterile enrichment indicated chemical oxidation as observed by Ottley et al. (1997); Supplementary Figure S5], the prominence of *Marinobacter*-related sequences and the isolation of *Marinobacter* sp. NP-6 from the rocks suggest that iron oxidation is supported in subsurface basalts (Supplementary Figure S2b). However, we noted the inconsistent results of *Marinobacter* abundance in this study and that of Meyer et al. (2016), which was possibly due to the use of different type of samples (basaltic rocks from our study versus crustal fluids from Meyer et al., 2016) and/or different primers (520F/802R from our study versus 967F/1046R from Meyer et al., 2016). As the microbial biomass in the samples was low (**Figure 1**), it was not yet clear if and how much the PCR primers have preference, and the number of 16S rRNA gene copies in different microbial cells may influence the 16S rRNA gene abundance in the samples (Vetrovsky and Baldrian, 2013). Therefore, the abundance of the bacterial groups might be under- or overrepresented through the present PCR sequencing methods.

## Conclusion

This study documents the potential for growth by subsurface basalt-associated microbial communities that possibly rely on iron oxidation for metabolic activity, and highlights nitrogen availability as an important parameter for stimulating growth in this ecosystem. This work supports earlier hypotheses that oxygen- and nitrate-fueled chemolithotrophic reactions are characteristic in low temperature ocean crust (Bach and Edwards, 2003; Edwards et al., 2012), distinct from high-temperature ridge flanks where methane and hydrogen seem to be major energy sources (Lever et al., 2013). This study confirms the existence of a deep biosphere hosted in young and oxic subsurface oceanic crust, filling a prior gap in knowledge about the extent of life on Earth. Further work including *in situ* microbiological and geochemical measurements is needed to determine the variability in cell abundance and activity in the crustal subsurface biosphere, and to determine its impact on global biogeochemical cycles.

## Author Contributions

FW designed the experiments, performed the incubation experiments, analyzed the data and wrote the manuscript. XZ designed and performed the incubation and isolation experiments, analyzed the data and wrote the paper. XZ conducted the cell enumeration experiment. JF analyzed the data. BO analyzed the data and wrote the manuscript. WB and KE co-led the expedition, analyzed the data and wrote the manuscript. All authors commented on the manuscript.

## Conflict of Interest Statement

The authors declare that the research was conducted in the absence of any commercial or financial relationships that could be construed as a potential conflict of interest.
