# Supplementary material for: Nitrogen Stimulates the Growth of Subsurface Basalt-associated Microorganisms at the Western Flank of the Mid-Atlantic Ridge
Source: Front Microbiol. 2016 May 3;7:633. doi: 10.3389/fmicb.2016.00633 (PMC4853389; doi:10.3389/fmicb.2016.00633)
Supplement: Supplementary file 1 [file Data_Sheet_1.DOC]

**Supplementary Material**

**Nitrogen stimulates the growth of subsurface basalt-associated microorganisms at the western flank of the Mid-Atlantic Ridge**

**Xinxu Zhang^1,2^, Jing Fang^1,2^, Wolfgang Bach^3^, Katrina J. Edwards^4#^, Beth N. Orcutt^5^, Fengping Wang^1,2*^**

^1^ State Key Laboratory of Microbial Metabolism, School of Life Sciences and Biotechnology, Shanghai Jiao Tong University, Shanghai 200240, People’s Republic of China.

^2^ State Key Laboratory of Ocean Engineering, School of Naval Architecture, Ocean and Civil Engineering, Shanghai Jiao Tong University, Shanghai 200240, People’s Republic of China.

^3^ MARUM Center for Marine Environmental Sciences & Department of Geosciences, University of Bremen, Bremen 28359, Germany.

^4^University of Southern California, Department of Biological Sciences, Los Angeles, California 90089, USA

^5^ Bigelow Laboratory for Ocean Sciences, East Boothbay, Maine 04544, USA

*** Correspondence:** Fengping Wang, State Key Laboratory of Microbial Metabolism, School of Life Sciences and Biotechnology, Shanghai Jiao Tong University, 800 Dongchuan Road, Shanghai, 200240, People’s Republic of China. Email: fengpingw@sjtu.edu.cn

# deceased

**Sample collection and contamination tests**

The information is already published in the Methods section of IODP Expedition 336 reports and reprinted here.

To examine potential contamination of hard rock and sediment core samples during drilling, slurries of yellow-green fluorescent microspheres (Fluoresbrite Carboxylate Microspheres; Polysciences, Inc., 15700) were sealed in plastic bags and placed inside the core catcher prior to deployment of the core barrel according to standard protocol . Immediately following delivery of basalt cores on deck, only large (>10 cm in length) intact whole-round pieces of rock samples were selected for microbiological study. Combusted aluminum foil or sterile Whirl-Pak bag were used for transport of samples to the microbiology laboratory. Whole-round rock pieces were then transferred to sterile Whirl-Pak bags containing 10 mL of sterile filtered seawater for gentle rinsing and removal of any microspheres and other contaminates. The rinsing process was repeated three times and the rinse was collected into a 15 mL conical vial and stored at 4 °C until processing. Fluorescent microspheres in the rinse were quantified by epi-fluorescence microscopy with a blue filter set according to standard protocol . Both basalt and sediment samples used in this study showed no microsphere after the third wash, indicating that samples passed the contamination test and were generally decontaminated. An exception was that only one microsphere was detected from sample 2R-2E in the final wash, which was possibly contaminated during drilling. Next, rocks were transferred to a flame-sterilized rock processing box and broken into smaller pieces using flame-sterilized chisels and forceps. Only interior pieces of a rock sample was selected for microbiological study as had been shown by multiple studies that the interiors of rock cores are generally free from contamination . They were further ground into sand-sized fractions with a flame-sterilized steel mortar and pestle and used for subsequent enrichment experiments. All sample handlers wore gloves to avoid contamination during the whole process. To avoid potential contamination and cell death during sample transportation, the enrichment experiments were directly performed on shipboard. Fluorescent microspheres in the basalts and their enrichments were further checked by epi-fluorescence microscopy on delivering to home laboratory. No microsphere was detected from all of the samples (including 2R-2E), which suggested that potential contamination during drilling and experimentation was minimal.

**Sediment incubation experiments**

Approximately 20 cm^3^ sediment sample from the interior of a whole round core was mixed with 40 mL filtered seawater in 100 mL glass bottles. Core 1H-2 enrichments were supplemented with a combination of ammonium chloride and one carbon substrate (sodium acetate, sodium bicarbonate, methane or methanol). Core 6H-8 enrichments were supplemented with the same combination as 1H-2 samples except the substitution of sodium nitrate for ammonium chloride (see Table 2 for substrate concentrations). A control treatment was also conducted with no carbon or nitrogen amendment. All bottles were capped with butyl rubber stoppers with filter-sterilized air in the headspace, and all enrichments were incubated at 10 °C and kept in the dark.

**Cell extraction and enumeration**

100 μL of preserved slurry was mixed with 500 μL acetate buffer (0.43 M glacial acetic acid, 0.43 M sodium acetate, dissolved in 3.5% NaCl solution) to dissolve carbonates for 2 h. Carbonate-free slurries were centrifuged for 5 min at 3,000 × *g*, and the supernatant was removed and kept for counting. The remaining pellet was then resuspended with 500 μL sodium chloride : formalin solution (3.5% NaCl, 3% formalin), followed by 50 μL detergent mix (100 mM disodium EDTA dihydrate, 100 mM sodium pyrophosphate decahydrate, 1 % Tween 80, in 3.5% NaCl solution) and 50 μL methanol. After vortexing for 2 h, a cushion of 500 μL 50% (wt/vol) Nycodenz® was layered below the slurry with a 12-gauge needle syringe and centrifuged at 1,500 × *g* for 10 min. The supernatant was then transferred to a separate tube, discarding the remaining Nycodenz® layer. The remaining pellet was resuspended in 500 μL sodium chloride solution, 50 μL detergent mix, and 50 μL methanol. The vial was sonicated at 20 W in an ice-water bath for 3×10 s with a 20 s interval. Addition of Nycodenz® followed by centrifugation was repeated as described above and the supernatant was transferred to a separate tube. The three supernatants were filtered through a 0.22-μm mesh GTBP membrane (Millipore) and stained with SYBR® Green I solution (1:40 vol/vol SYBR® Green I in 1×Tris-EDTA buffer) for 20 min. The stain solution was removed, the filter was placed onto a glass slide, and 25 μL 10% glycerine was added as an antifade agent. Cells were counted at 1000× magnification using an epi-fluorescence microscope (Nikon, ECLIPSE 90i, Japan) with a blue filter set. Each sample was extracted and counted in triplicate. An average of 135 fields of view was counted for each membrane. In addition, quantitative PCR analysis of the bacterial 16S rRNA gene was performed on DNA extracts from the 10R-1B samples as an additional measure of cell abundance. Gene abundance was quantified using primers 341f and 519r (Table S1) using SYBR® Premix Ex Taq II kit (TaKaRa) with a thermal cycle of 95 °C for 15 min, 35 cycles of 95 °C for 15 s, 58 °C for 30 s, and 72 °C for 30 s.

**Nucleic acid extraction, amplification, and sequencing of 16S rRNA gene**

DNA was extracted from the 30R-1A samples using FastDNA™ SPIN Kit for Soil (MP Biomedicals, Santa Ana, USA) according to manufacturer instructions with few modifications. For the original rock samples, approximately 0.5 g rock sample was ground into powder with a double flame-sterilized mortar and pestle. 5 μg of clean, UV irradiated poly-dIdC (Sigma-Aldrich) was added to increase the yield of DNA for low-biomass rock samples according to . For enrichments, 1 mL slurry was added to a Lysing Matrix E tube (FastDNA™ SPIN Kit for Soil, MP Biomedicals, USA) and centrifuged at 12,000×*g* for 2 min. The supernatant was discarded and the pellet was resuspended with sodium phosphate buffer (FastDNA™ SPIN Kit for Soil, MP Biomedicals, USA). The V4 region of the bacterial 16S rRNA gene was amplified using multi-tag primers 520F and 802R (Table S1) and generated ~240 bp amplicons. The PCR program involved an initial denaturation step at 95 °C for 10 min, followed by 30 cycles of denaturation at 95 °C for 45 s, annealing at 55 °C for 1 min, and extension at 72 °C for 1 min, and a final extension at 72 °C for 5 min. The DNA blank extraction was performed without template sample and processed with the same DNA extraction and PCR amplification kits as the basalt samples. DNA extraction and PCR amplification were considered free of contamination if no target PCR band around 240 bp was seen on the agarose gel both for the blank DNA extraction and PCR negative control. The 16S rRNA gene amplicons containing unique 8-mer barcodes used for each sample were pooled with equal concentration, and sequenced on an Illumina MiSeq platform using 2 × 250 bp cycles and MiSeq Reagent Kit v2 (500 cycle, Illumina, USA) according to manufacturer instructions. Raw reads were removed if contained 50 bp continuous fragment with an average quality score less than 30 and/or any ambiguities. Filtered reads were merged together using FLASH . Merged sequences were further removed if they contained more than six homopolymers and/or any ambiguities, or sequence length was <200 bp. Clean sequences were demultiplexed using the QIIME software pipeline with a mapping file containing the sample ID, barcode and primer sequence. No archaeal 16S rRNA gene sequences were obtained although different archaeal primers were tested (see Table S1), suggesting a low abundance of archaea in the sample.

**Assessment results for potential contaminating sequences**

Considering the potential for contamination of the rocks during sampling , and the potential for spurious sequences from commercial kits used for DNA extract preparation and sequencing of relatively low biomass samples , we included procedural controls during collection and preparation, and we screened the resulting sequence library according to pipelines developed previously . Drilling contamination was assessed through the application of microsphere tracers in the drilling fluids; none were detected in the samples used in this study, as reported elsewhere . No sequences of common surface seawater bacteria, like Cyanobacteria, were recovered in the sequence library, as discussed below—another indication that contamination with surface-seawater derived drilling fluids did not occur. Moreover, comparative taxonomic analysis of the sequences from this study with those from the drilling mud sample revealed distinct separation of rock-hosted microorganisms from drilling mud influence . Spurious sequences from commercial kits were identified through a comparative phylogenetic analysis of the sequence library to a database of known contaminants with sequence coverage over the same span of the 16S rRNA gene as the sample library . As suggested by , any sequences that demonstrated >98% sequence similarities to the contaminant database were flagged as possible contaminants. Seven OTUs (representing 0.02 – 11.3% of the total quality-screened sequences, see Figures 3, S7, S8, S10) were identified as having 98% or greater sequence similarity to known sequencing contaminants (Table S4). These included the genera *Acinetobacter*, *Agrobacterium*, *Bradyrhizobium*, *Curvibacter*, *Ralstonia*, *Sphingomonas* and *Stenotrophomonas*. However, cultures of some of these same microbial groups have recently been reported from basalts and sediment from this same expedition , in particular from the genera *Ralstonia* and *Sphingomonas*, indicating that removal of these flagged sequences from the library may not be warranted. Thus, sequences identified as possible sequence contaminants are retained in the library but are highlighted in the overall description of the community structure (Figure S6).


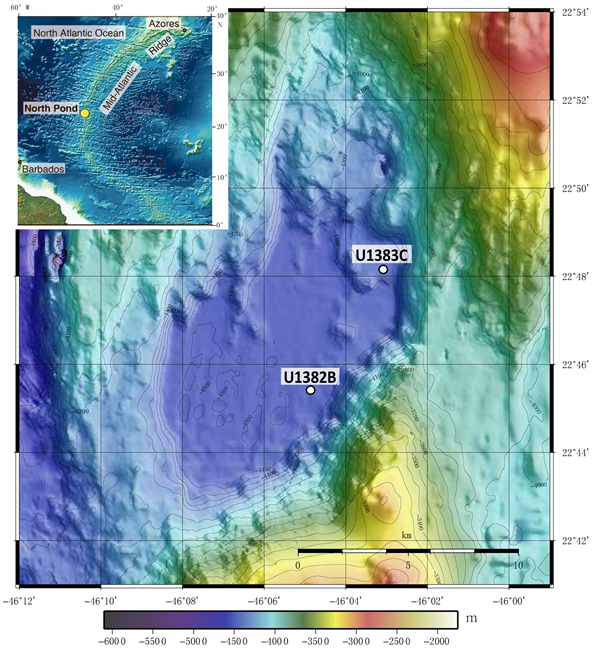


**Figure S1. Location map and sampling sites.** The yellow dot in the upper left shows the location of North Pond on the western flank of Mid-Atlantic Ridge. The sampling sites are indicated with white dots in this study. The color scale at the bottom indicates the water depth. Map modified from the IODP Expedition 336 Preliminary Report .

**
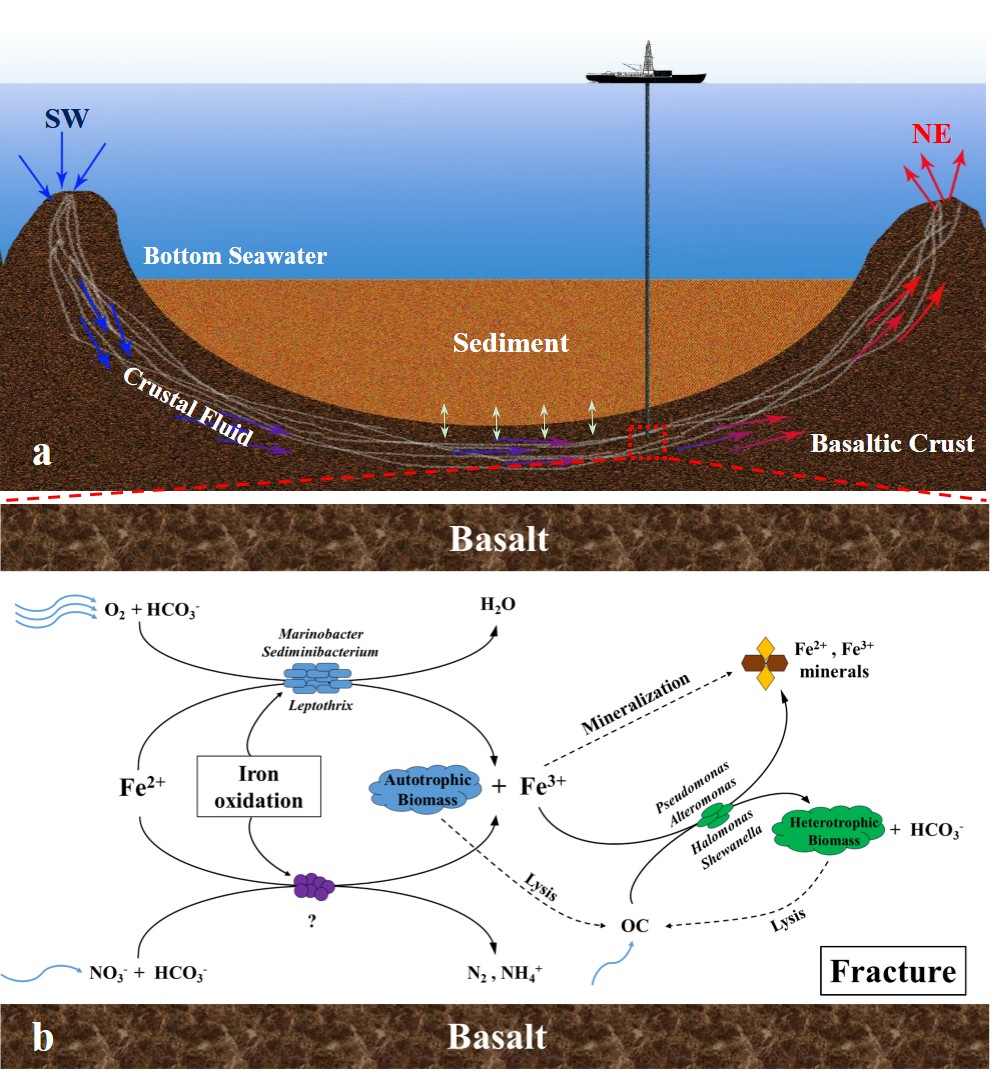
Figure S2. Schematic diagram of North Pond subsurface crustal biosphere and microbial iron and carbon cycling within the basalt fractures.** **(a)** Rock samples were collected at the basaltic basement (72-304 mbsf) under a ~70 m thick sediment layer (not to scale). Originating from the southern or western rim, crustal fluids circulates through the permeable basaltic basement and discharges along the northern and eastern edges . SW, southwest. NE, northeast. OC, organic carbon. Blue arrows indicates cold fluids (~5 °C). Red arrows indicates warm fluids (up to ~25 °C). **(b)** Crustal fluids bring O_2_, HCO_3_^-^, NO_3_^-^ and organic carbon with bottom seawater signatures and penetrate into basalt fractures where microorganisms reside. The blue line with arrow indicates crustal fluid. The dominant microorganisms in the basement are likely supported by lithotrophic iron oxidation (with oxygen or nitrate reduction) for metabolic activities. The organic carbon produced through lithotrophic activities and brought by circulating crustal fluids may further support the growth of heterotrophic bacteria in the basalt.


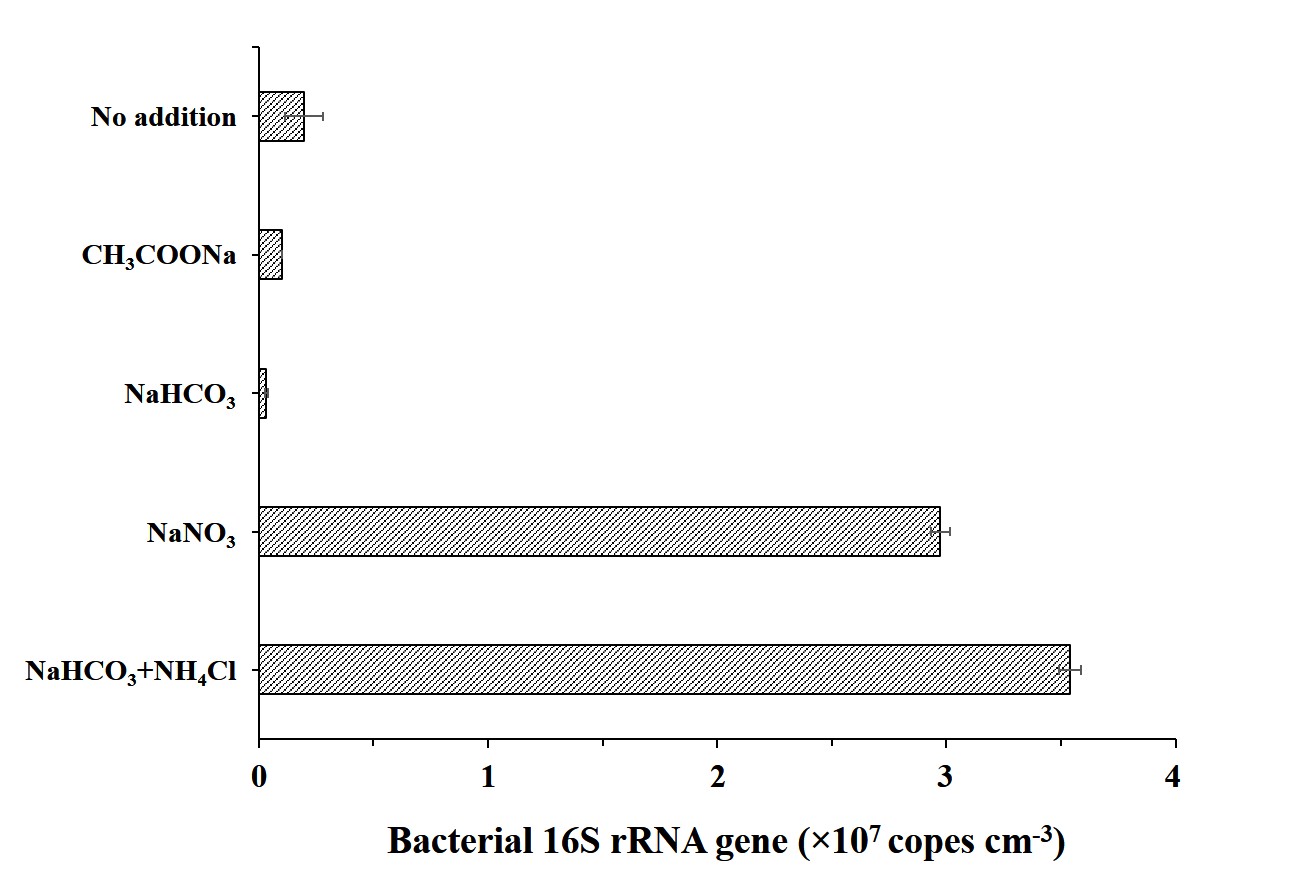


**Figure S3. Bacterial 16S rRNA gene copes of 10R-1B enrichments as measured by quantitative PCR.** Cell enumeration results (Figure 1) were confirmed by qPCR method using 10R-1B enrichments as an example. See Materials and Methods section for more details.


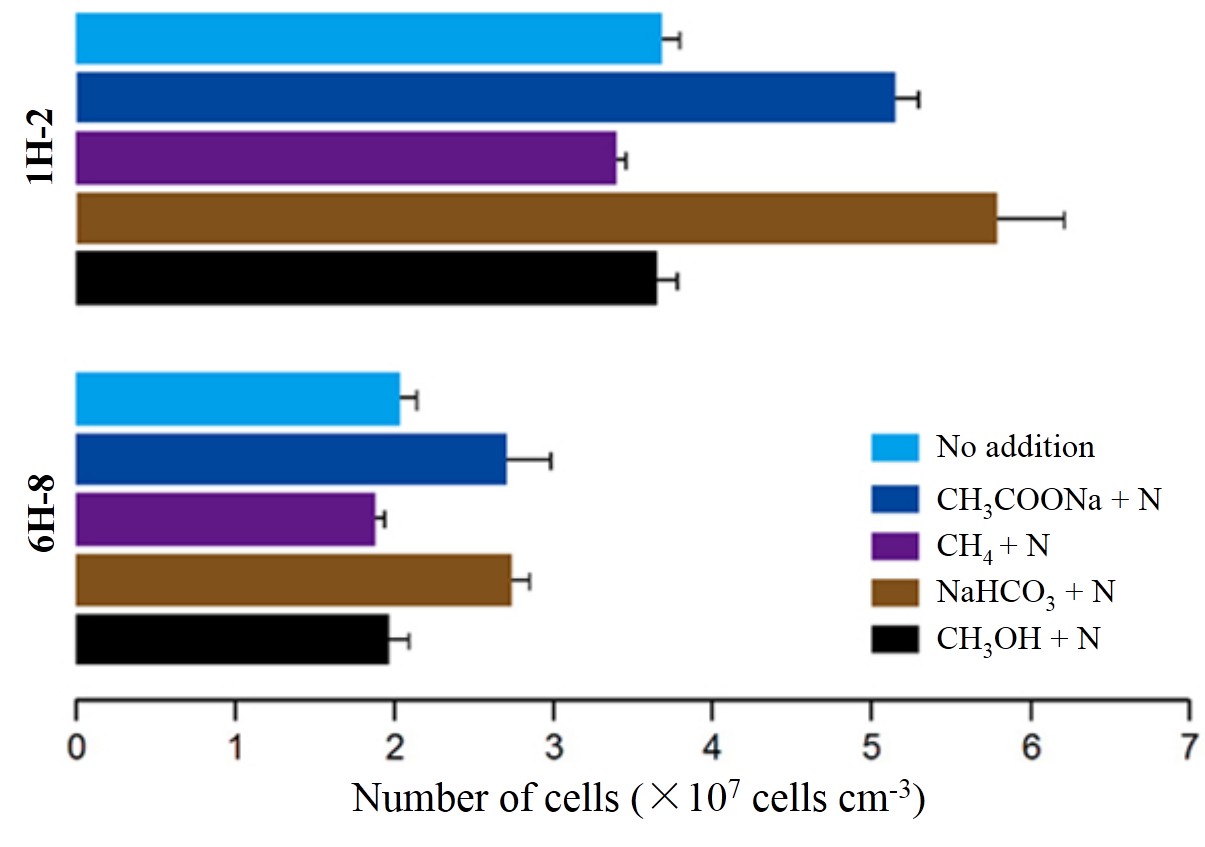


**Figure S4. Sediment cell counts after 6 months of incubation with carbon and nitrogen substrates.** Two sections of sediment from Core U1382B are incubated and counted, 1H-2 (1.65 mbsf) and 6H-8 (49.88 mbsf). The enrichments of different substrates addition are shown with different color bars. N represents NH_4_Cl for 1H-2 (except CH_4_ + NaNO_3_) and NaNO_3_ for 6H-8 respectively. The “No addition” are negative control incubations without any added substrate.


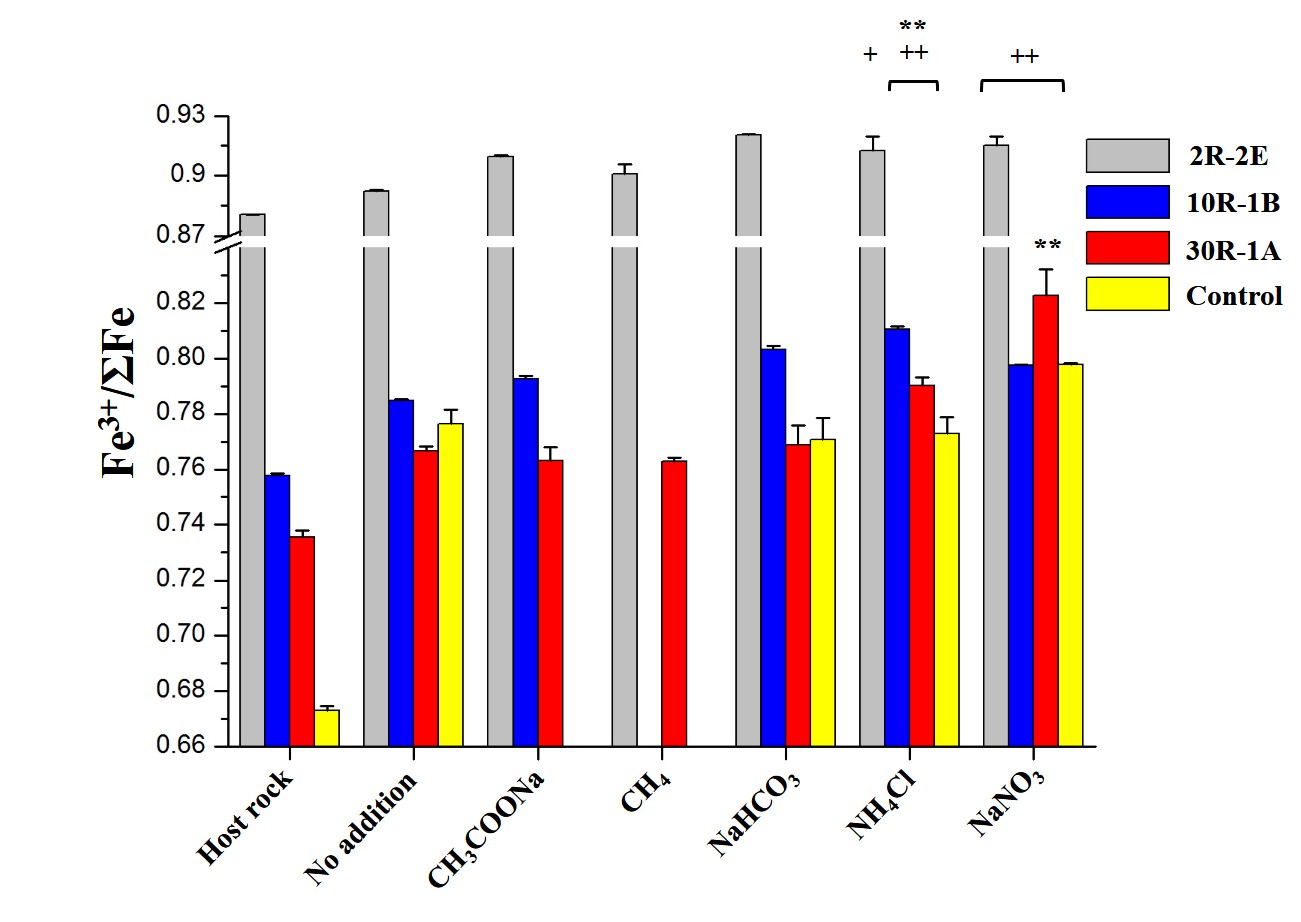


**Figure S5. Fe^3+^/ΣFe ratios of basaltic rocks and enrichments.** The Fe^3+^/ΣFe ratio indicates the oxidation state of iron in the medium. Higher Fe^3+^/ΣFe ratio indicates that more Fe^2+^ are oxidized to Fe^3+^ proportionally. The “Control” indicates 3R-4B enrichments which were double autoclaved and incubated at the same condition to test the extent of abiotic iron oxidation. ΣFe = Fe^2+^ + Fe^3+^. + P<0.05 and ++ P<0.01 versus No addition, ****** P<0.01 versus NaHCO_3_ by analyses of variance.


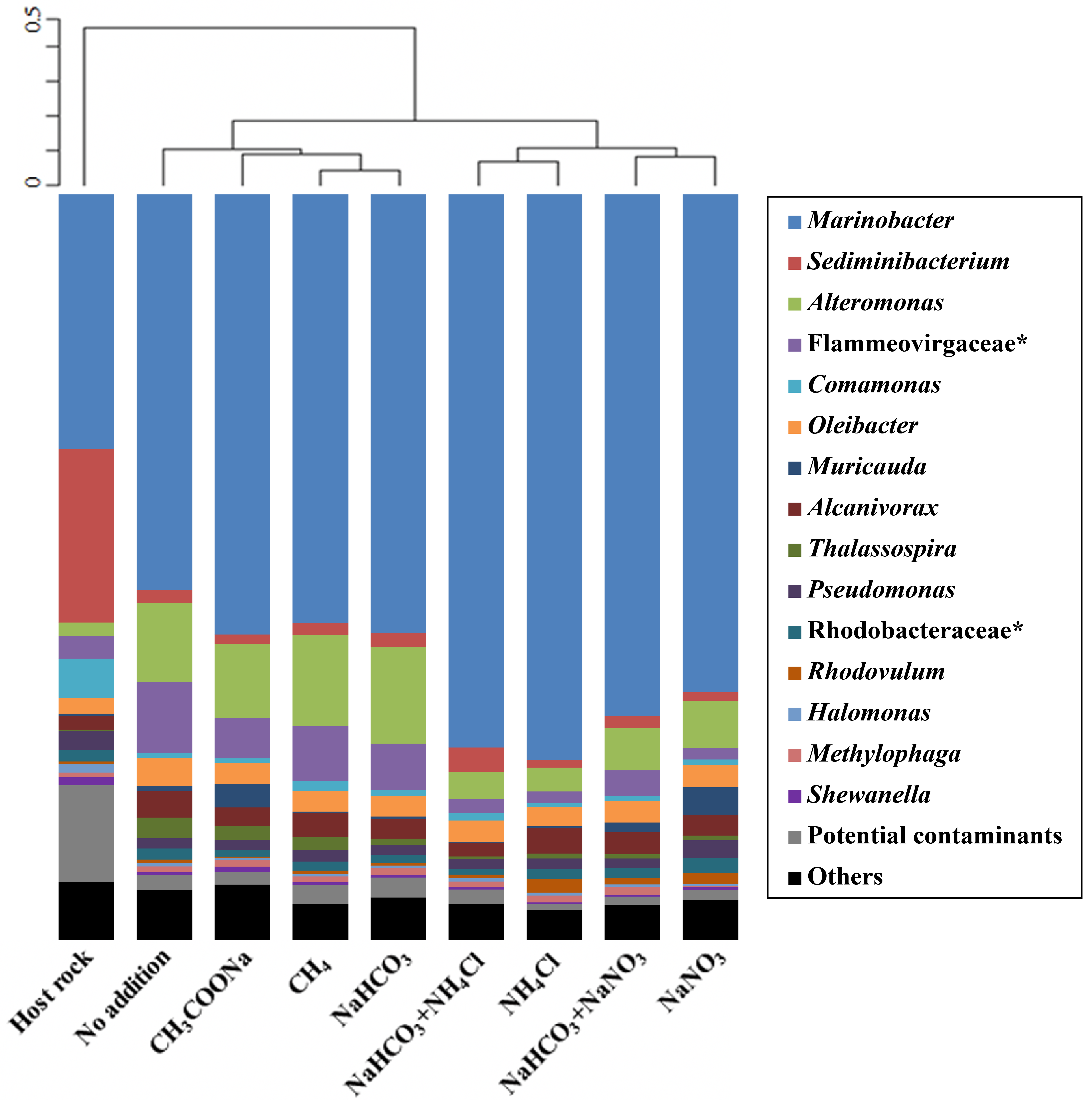


**Figure S6. Bacterial community composition and hierarchical clustering of 30R-1A host rock and enrichments based on 16S rRNA gene sequence abundance.** The sequence data of 30R-1A host rock were retrieved from . Color bars indicate the percentage of sequences in each designated genus, with asterisks symbol indicating OTUs that could not be assigned to genus level in the Greengenes 16S rRNA gene database. Genera <1% abundance are grouped into "Others", and sequences identified as potential sequence contaminants from commercial kits (i.e. *Acinetobacter*, *Agrobacterium*, *Bradyrhizobium*, *Curvibacter*, *Ralstonia*, *Sphingomonas* and *Stenotrophomonas*) are grouped into "Potential contaminants". Hierarchical cluster dendrogram of microbial communities are based on Bray-Curtis distance matrix. The scale bar indicates distance in length.


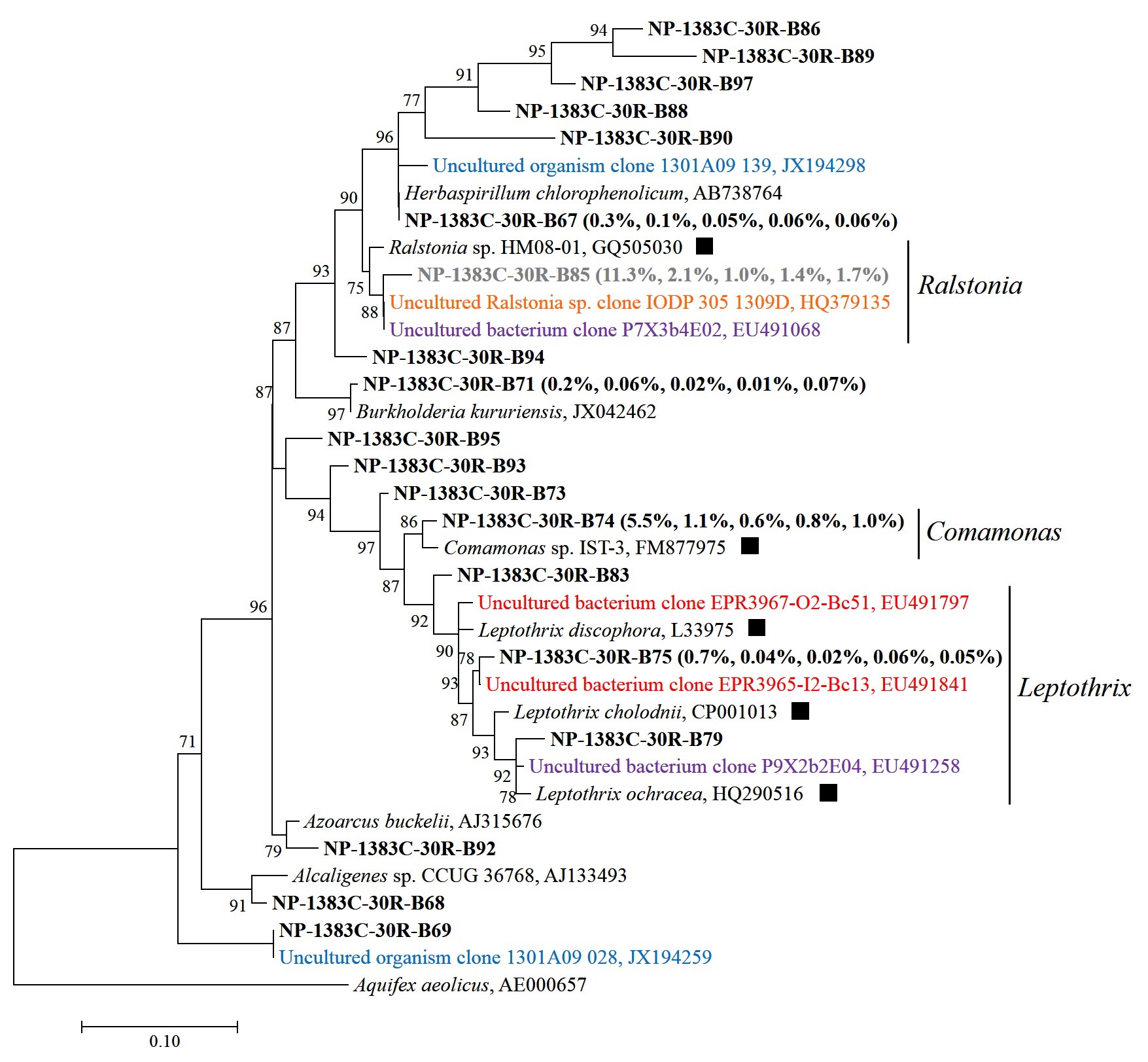


**Figure S7. Phylogenetic tree of Betaproteobacteria related 16S rRNA gene sequences from host rock sample 30R-1A in comparison to sequences from cultivated species and other environmental studies.** The sequence data of 30R-1A host rock are retrieved from , and are highlighted in bold font. The numbers in parentheses indicate percent abundance of the reads clustered in genera followed by the order “Host rock” “NaHCO_3_ + NH_4_Cl” “NH_4_Cl” “NaHCO_3_ + NaNO_3_” “NaNO_3_”, respectively. A representative sequence from sample 30R-1A for each genus is shown due to the high sequence number. Sequences from isolates or other environmental studies identified by Genbank accession number. Filled squares indicate known iron-oxidizing bacteria. Sequences retrieved from Atlantis Massif are in orange, East Pacific Rise in red, Loihi Seamount in purple, Juan de Fuca Ridge flank in blue, crustal fluids of Costa Rica Rift flank in brown. Potential kit contaminating sequences are in grey. The number at each branch indicates local support value, with only values >70 shown. The 16S rRNA gene of *Aquifex aeolicus* (AE000657) is used as outgroup. The scale bar indicates 0.1 nucleotide substitutions per site.


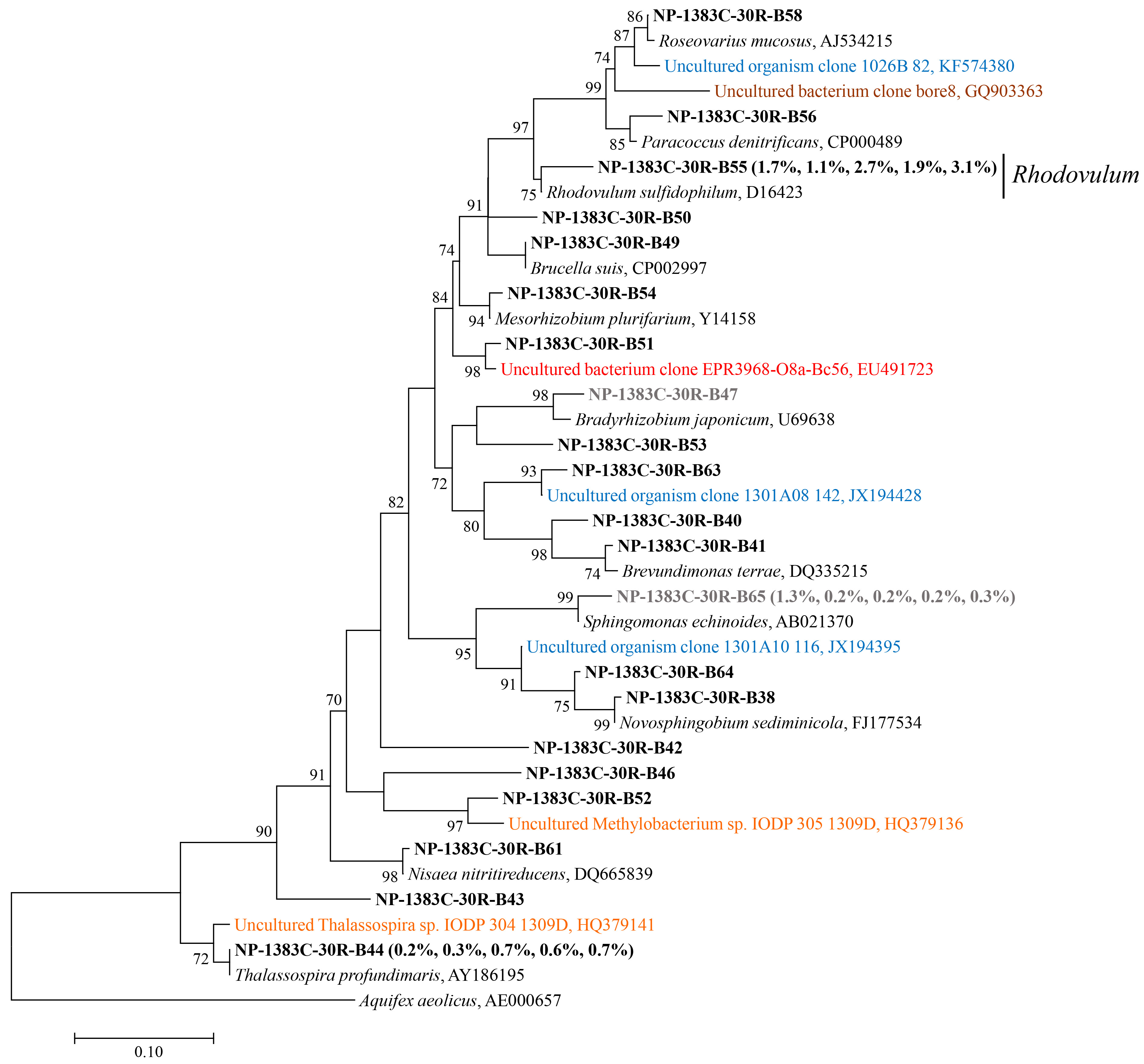


**Figure S8. Phylogenetic tree of Alphaproteobacteria related 16S rRNA gene sequences from host rock sample 30R-1A in comparison to sequences from cultivated species and other environmental studies.** The sequence data of 30R-1A host rock are retrieved from , and are highlighted in bold font. The numbers in parentheses indicate percent abundance of the reads clustered in genera followed by the order “Host rock” “NaHCO_3_ + NH_4_Cl” “NH_4_Cl” “NaHCO_3_ + NaNO_3_” “NaNO_3_”, respectively. A representative sequence from sample 30R-1A for each genus is shown due to the high sequence number. Sequences from isolates or other environmental studies identified by Genbank accession number. Sequences retrieved from Atlantis Massif are in orange, East Pacific Rise in red, Loihi Seamount in purple, Juan de Fuca Ridge flank in blue, crustal fluids of Costa Rica Rift flank in brown. Potential kit contaminating sequences are in grey. The number at each branch indicates local support value, with only values >70 shown. The 16S rRNA gene of *Aquifex aeolicus* (AE000657) is used as outgroup. The scale bar indicates 0.1 nucleotide substitutions per site.


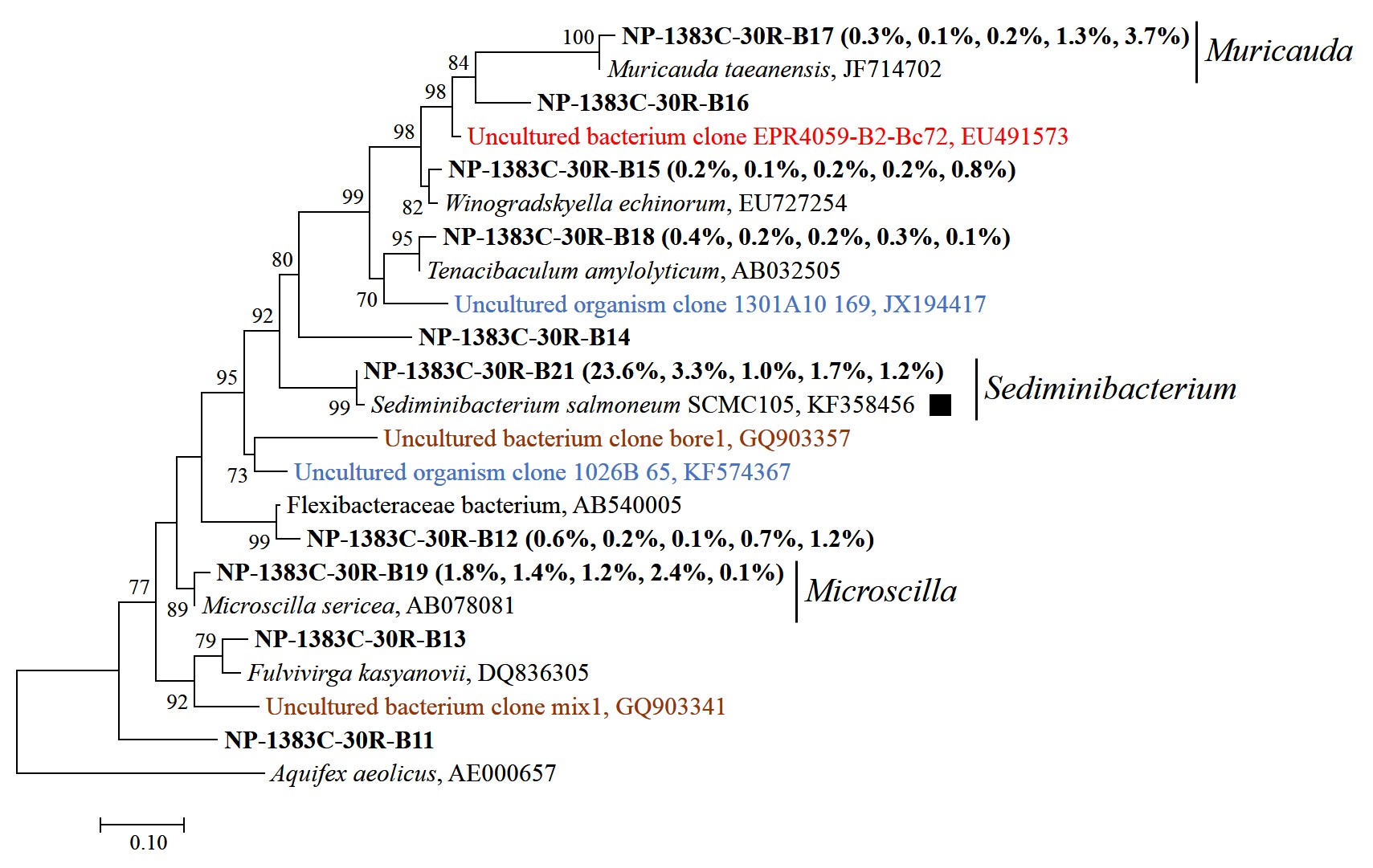


**Figure S9. Phylogenetic tree of Bacteroidetes related 16S rRNA gene sequences from host rock sample 30R-1A in comparison to sequences from cultivated species and other environmental studies.** The sequence data of 30R-1A host rock are retrieved from , and are highlighted in bold font. The numbers in parentheses indicate percent abundance of the reads clustered in genera followed by the order “Host rock” “NaHCO_3_ + NH_4_Cl” “NH_4_Cl” “NaHCO_3_ + NaNO_3_” “NaNO_3_”, respectively. A representative sequence from sample 30R-1A for each genus is shown due to the high sequence number. Sequences from isolates or other environmental studies identified by Genbank accession number. Filled squares indicate known iron-oxidizing bacteria. Sequences retrieved from Atlantis Massif are in orange, East Pacific Rise in red, Loihi Seamount in purple, Juan de Fuca Ridge flank in blue, crustal fluids of Costa Rica Rift flank in brown. The number at each branch indicates local support value, with only values >70 shown. The 16S rRNA gene of *Aquifex aeolicus* (AE000657) is used as outgroup. The scale bar indicates 0.1 nucleotide substitutions per site.


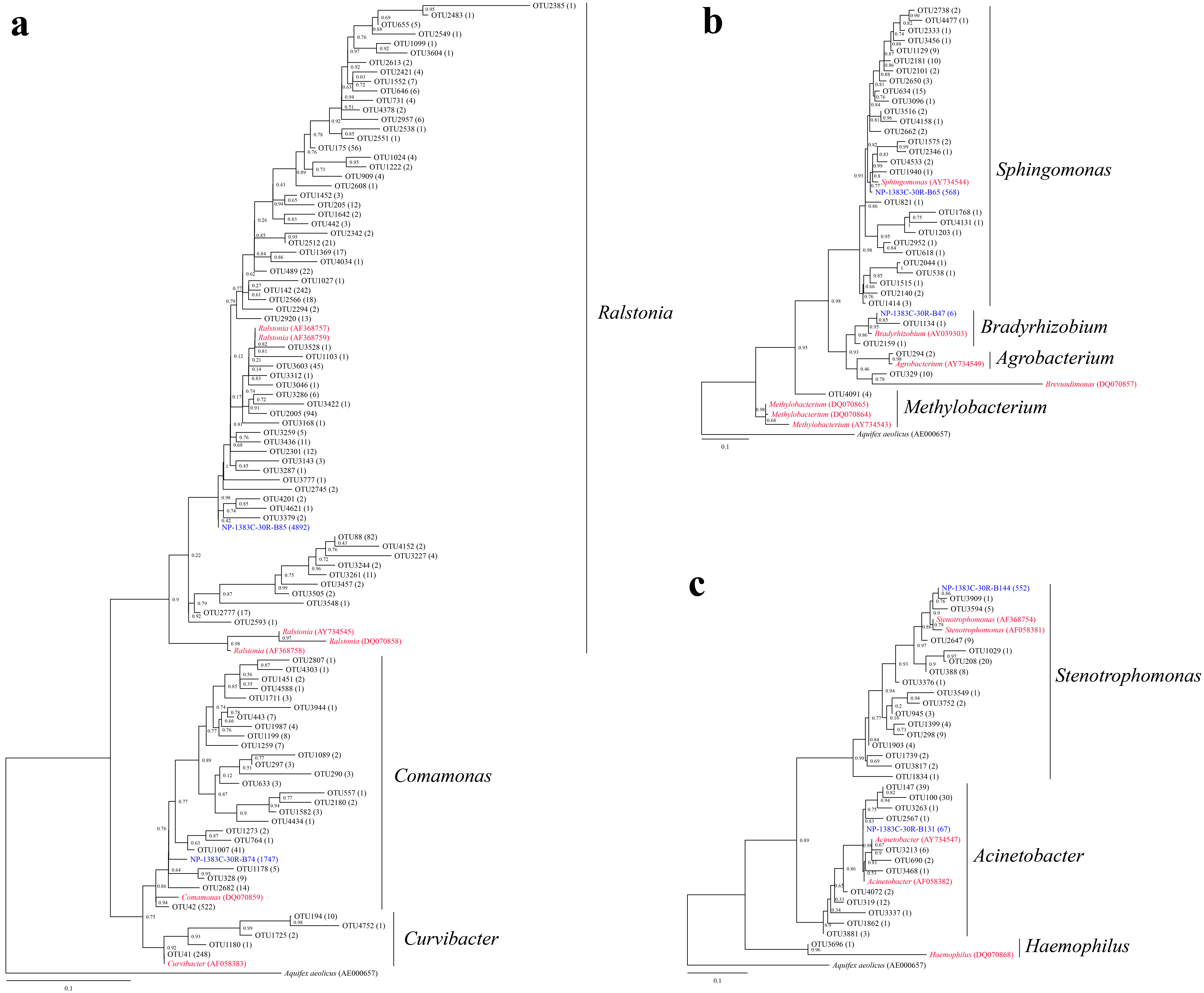


**Figure S10. Comparative phylogenetic analysis of the suspected contaminating sequences from 30R-1A against sequences from the low-biomass contaminant database.** The sequence data of 30R-1A host rock are retrieved from . A representative set of sequences from 30R-1A and all of its enrichments for each OTU are shown due to the high sequence number. (a) Betaproteobacterial related OTUs. (b) Alphaproteobacterial related OTUs. (c) Gammaproteobacterial related OTUs. Sequences from the low-biomass contaminant database are in red, which are retrieved from , and . Sequences are in blue if shown in Figures 3, S7, S8. The number in parentheses indicates total sequences of the designated OTU from 30R-1A and all of its enrichments. The 16S rRNA gene of *Aquifex aeolicus* (AE000657) is used as outgroup. The scale bar indicates 0.1 nucleotide substitutions per site.


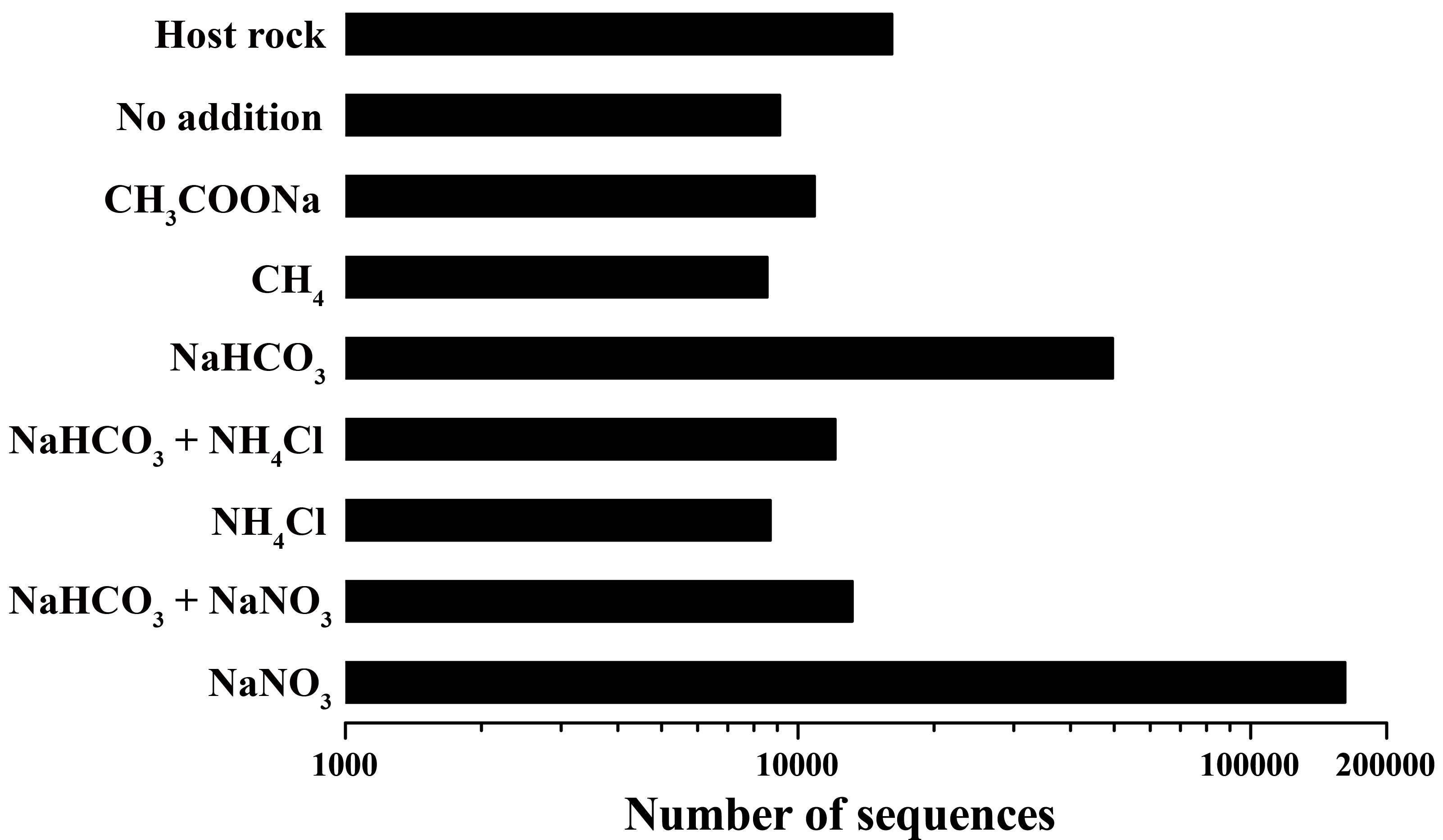


**Figure S11. Summary of bacterial 16S rRNA gene sequencing from 30R-1A sample prior to and after incubation for six months.** The sequence data of 30R-1A host rock are retrieved from . The number of sequences are after sequence quality filtering and chimera removal. The x-axis is log10 scaled.

**Table S1. Bacterial and Archaeal primer sequences tested in this study.**

| **Primer name** | **Primer sequences (5’-3’)** | **Reference** |
| --- | --- | --- |
| 520F^1,3^ | XXXXXXXX - AYTGGGYDTAAAGNG |  |
| 802R^1,3^ | TACNVGGGTATCTAATCC |
| U519F^2^ | YMGCCRCGGKAAHACC |
| Arch806R^2^ | CTACNSGGGTMTCTAAT |
| A2Fa^2^ | TTCCGGTTGATCCYGCCRGA |  |
| 519R^2^ | GWATTACCGCGGCKGCTG |
| Arch21F^2^ | TTCCGGTTGATCCYGCGGA |  |
| Arch958R^2^ | YCCGGCGTTGMTCCAATT |
| 341f^1,4^ | CCTACGGGWGGCWGCA |  |
| 519r^1,4^ | TTACCGCGGCKGCTG |

1. Bacterial primer; 2. Archaeal primer; 3. Primer for sequencing, X indicates the barcode sequence; 4. Primer for qPCR.

**Table S2. Cell abundance and cell division times after incubation for six months.**

| **Substrate** | **Number of cells (×10^5^ cells cm^-3^)** | | | |
| --- | --- | --- | --- | --- |
|  | 2R-2E | 10R-1B | 30R-1A | Average |
| No addition | 1.95 ± 0.34 (1.9) | 0.98 ± 0.34 (0.0) | 7.23 ± 0.34 (2.1) | 3.39 (1.3) |
| CH_3_COONa | 8.98 ± 1.22 (4.1) | 9.37 ± 0.59 (3.1) | 15.62 ± 2.95 (3.5) | 11.32 (3.6) |
| CH_4_ | 11.33 ± 1.47 (4.4) | NA | 13.47 ± 2.11 (3.0) | 12.40 (3.7) |
| NaHCO_3_ | 2.54 ± 0.68 (2.2) | 1.37 ± 0.34 (0.3) | 6.05 ± 1.47 (1.9) | 3.32 (1.5) |
| NaHCO_3_ + NH_4_Cl | 278.09 ± 12.38 (9.0) | 58.00 ± 2.11 (5.7) | 47.06 ± 3.23 (4.9) | 127.72 (6.5) |
| NH_4_Cl | 53.31 ± 4.42 (6.6) | 29.88 ± 0.59 (4.7) | 54.68 ± 3.77 (5.1) | 45.96 (5.5) |
| NaHCO_3_ + NaNO_3_ | 47.06 ± 5.44 (6.4) | 66.40 ± 7.95 (5.9) | 32.22 ± 4.69 (4.3) | 48.56 (5.5) |
| NaNO_3_ | 48.24 ± 1.47 (6.5) | 34.76 ± 3.90 (5.0) | 39.45 ± 5.63 (4.6) | 40.82 (5.4) |

The number in parentheses indicates cell division times. Assumption: no cell death considered; cells reproduce by binary fission. NA indicates not applicable for lack of samples.

**Table S3. Ammonium and nitrate concentrations of basalt enrichments after six months of incubation.**

| **Substrate** | **Ammonium (mM)** | | | | **Nitrate (mM)** | | | |
| --- | --- | --- | --- | --- | --- | --- | --- | --- |
|  | 2R-2E | 10R-1B | 30R-1A | Control | 2R-2E | 10R-1B | 30R-1A | Control |
| No addition | 0.01 | 0.01* | 0.02 | 0.01 | 0.02 | 0.02 | 0.05 | 0.03 |
| CH_3_COONa | 0.01 | 0.01* | 0.02 | NA | 0.06 | 0.04 | 0.06 | NA |
| CH_4_ | 0.01 | NA | NA | NA | 0.03 | NA | NA | NA |
| NaHCO_3_ | 0.01 | 0.02* | 0.02 | 0.01 | 0.02 | 0.04 | 0.04 | 0.04 |
| NaHCO_3_ + NH_4_Cl | 0.76 | 0.78 | 0.86 | 1.52 | 0.03 | 0.03 | 0.04 | 0.04 |
| NH_4_Cl | 1.02 | 0.85* | 1.10 | NA | 0.05 | 0.05 | 0.03 | NA |
| NaHCO_3_ + NaNO_3_ | 0.09* | 0.03* | 0.02 | 0.02 | 1.97 | 2.77 | 2.48 | 3.06 |
| NaNO_3_ | 0.11 | 0.01* | 0.02 | NA | 2.15 | 2.64 | 2.55 | NA |

The “No addition” are negative control incubations without any added substrate. The “Control” is the sterile 3R-4B enrichments. * Nitrite is detected in trace amount (2 – 3 μM). NA indicates not applicable for lack of samples.

**Table S4. ClustalW Alignment of the suspected contaminating sequences in this study against sequences from the low-biomass contaminant database.**

| **Sequence ID from this study** | **Sequences from the**  **contaminant database** | **Accession No.** | **Identity (%)** |
| --- | --- | --- | --- |
| OTU41 (248) | *Curvibacter* | AF058383 | 100 |
| NP-1383C-30R-B131 (67) | *Acinetobacter* | AF058382 | 100 |
| NP-1383C-30R-B85 (4892) | *Ralstonia* | AF368757 | 99 |
| NP-1383C-30R-B65 (568) | *Sphingomonas* | AY734544 | 98.7 |
| OTU294 (2) | *Agrobacterium* | AY734549 | 98.7 |
| NP-1383C-30R-B47 (6) | *Bradyrhizobium* | AY039303 | 97.8 |
| NP-1383C-30R-B144 (552) | *Stenotrophomonas* | AF368754 | 97.8 |

The number in parentheses indicates total number of sequences in the designated OTU, including the host rock and all of its enrichments.

**Table S5**. Summary of microbial community abundance and composition from oceanic crust environments.

| **Site,**  ***rock type*** | **Cell density (cells cm^-3^)** | **Sample depth (mbsf^a^)** | **Water depth (m)** | **Temp. (°C)** | **Ref.** |
| --- | --- | --- | --- | --- | --- |
| North Pond,  *aphyric/phyric basalt* | 10^4^ - 10^5*^ *Bacteria* | 72-304 | 4,425-4,492 | 5-25 | This study |
| Atlantis Massif,  *gabbro & peridotite* | < 10^3*^  *Bacteria* | 62-1,391 | 1,645 | 14-102 | I |
| Juan de Fuca Ridge flank,  *crustal fluids from basalt* | 10^3^ - 10^4*^  *Varied* | 370 | 2,667 | 65 | II |
| Hawaii volcano,  *hyaloclastite* | ~ 10^5#^  *Archaea* | 1,336-1,404 | 0 | 15 | III |
| Loihi Seamount,  *basalt glass* | ~10^6&^  *Bacteria* | Se | 1,178-4,987 | ~2 | IV |
| East Pacific Rise,  *basalt glass* | 10^6^ - 10^9&^  *Bacteria* | Se | 2,516-2,674 | ~2 | V |
| Knipovich & Mohns Ridges,  *basalt glass* | 10^5^ - 10^6&^  *Bacteria* | Se | 2,500-3,390 | ~2 | VI |

a: mbsf, meters below seafloor. Se: seafloor exposed.

Cell enumeration methods: * direct counting, # amino acid deduced, & quantitative PCR

I: Mason et al., 2010; II: Jungbluth et al., 2013; III: Fisk et al., 2003; IV: Jacobson Meyers et al., 2014; V: Santelli et al., 2008; VI: Einen et al., 2008.

**References:**

Barton, H.A., Taylor, N.M., Lubbers, B.R., and Pemberton, A.C. (2006). DNA extraction from low-biomass carbonate rock: an improved method with reduced contamination and the low-biomass contaminant database. *J. Microbiol. Methods* 66**,** 21-31. doi: 10.1016/j.mimet.2005.10.005.

Caporaso, J.G., Kuczynski, J., Stombaugh, J., Bittinger, K., Bushman, F.D., Costello, E.K., Fierer, N., Pena, A.G., Goodrich, J.K., Gordon, J.I., Huttley, G.A., Kelley, S.T., Knights, D., Koenig, J.E., Ley, R.E., Lozupone, C.A., McDonald, D., Muegge, B.D., Pirrung, M., Reeder, J., Sevinsky, J.R., Tumbaugh, P.J., Walters, W.A., Widmann, J., Yatsunenko, T., Zaneveld, J., and Knight, R. (2010). QIIME allows analysis of high-throughput community sequencing data. *Nat. Methods* 7**,** 335-336. doi: 10.1038/nmeth.f.303.

DeLong, E.F. (1992). Archaea in coastal marine environments. *Proc. Natl. Acad. Sci. U.S.A.* 89**,** 5685-5689. doi: 10.1073/pnas.89.12.5685.

Expedition 327 Scientists (2011). Methods. *In* Fisher, A.T., Tsuji, T., Petronotis, K., and the Expedition 327 Scientists, *Proc. IODP,* **327**: Tokyo (Integrated Ocean Drilling Program Management International, Inc.). doi:10.2204/iodp.proc.327.102.2011.

Expedition 330 Scientists (2012). Methods. *In* Koppers, A.A.P., Yamazaki, T., Geldmacher, J., and the Expedition 330 Scientists, *Proc. IODP*, 330: Tokyo (Integrated Ocean Drilling Program Management International, Inc.). doi:10.2204/iodp.proc.330.102.2012.

Expedition 336 Scientists (2012a). Methods in *Proc. IODP, 336*. (eds Edwards KJ, Bach W, Klaus A, and the Expedition 336 Scientists) (Integrated Ocean Drilling Program Management International, Inc.). doi: 10.2204/iodp.proc.336.102.2012.

Expedition 336 Scientists (2012b). Mid-Atlantic Ridge microbiology: Initation of long-term coupled microbiological, geochemical, and hydrological experimentation within the seaﬂoor at North Pond, western ﬂank of the Mid-Atlantic Ridge. *IODP Prel. Rep.* **336**. doi: 10.2204/iodp.pr.336.2012.

Hirayama, H., Abe, M., Miyazaki, J., Sakai, S., Nagano, Y., and Takai, K. (2015). Data report: cultivation of microorganisms from basaltic rock and sediment cores from the North Pond on the western flank of the Mid-Atlantic Ridge, IODP Expedition 336. *In* Edwards, K.J., Bach, W., Klaus, A., and the Expedition 336 Scientists, *Proc. IODP,* **336**: Tokyo (Integrated Ocean Drilling Program Management International, Inc.). doi:10.2204/iodp.proc.336.204.2015.

Jorgensen, S.L., Hannisdal, B., Lanzén, A., Baumberger, T., Flesland, K., Fonseca, R., Øvreås, L., Steen, I.H., Thorseth, I.H., Pedersen, R.B., and Schleper, C. (2012). Correlating microbial community profiles with geochemical data in highly stratified sediments from the Arctic Mid-Ocean Ridge. *Proc. Natl. Acad. Sci. U.S.A.* 109**,** E2846–E2855. doi: 10.1073/pnas.1207574109.

Kim, M., Morrison, M., and Yu, Z.T. (2011). Evaluation of different partial 16S rRNA gene sequence regions for phylogenetic analysis of microbiomes. *J. Microbiol. Methods* 84**,** 81-87. doi: 10.1016/j.mimet.2010.10.020.

Kulakov, L.A., McAlister, M.B., Ogden, K.L., Larkin, M.J., and O'Hanlon, J.F. (2002). Analysis of Bacteria Contaminating Ultrapure Water in Industrial Systems. *Appl. Environ. Microbiol.* 68**,** 1548-1555. doi: 10.1128/aem.68.4.1548-1555.2002.

Lever, M.A., Alperin, M., Engelen, B., Inagaki, F., Nakagawa, S., Steinsbu, B.O., Teske, A., and Sci, I.E. (2006). Trends in basalt and sediment core contamination during IODP Expedition 301. *Geomicrobiol. J.* 23**,** 517-530. doi: 10.1080/01490450600897245.

Magoč, T., and Salzberg, S.L. (2011). FLASH: fast length adjustment of short reads to improve genome assemblies. *Bioinformatics* 27**,** 2957-2963. doi: 10.1093/bioinformatics/btr507.

Meyer, J.L., Jaekel, U., Tully, B.J., Glazer, B.T., Wheat, C.G., Lin, H.-T., Hsieh, C.-C., Cowen, J.P., Hulme, S.M., Girguis, P.R., and Huber, J.A. (2016). A distinct and active bacterial community in cold oxygenated fluids circulating beneath the western flank of the Mid-Atlantic ridge. *Sci. Rep.* 6**,** 22541. doi: 10.1038/srep22541.

Orcutt, B.N., Wheat, C.G., Rouxel, O., Hulme, S., Edwards, K.J., and Bach, W. (2013). Oxygen consumption rates in subseafloor basaltic crust derived from a reaction transport model. *Nat. Commun.* 4**,** 2539. doi: 10.1038/ncomms3539.

Salter, S.J., Cox, M.J., Turek, E.M., Calus, S.T., Cookson, W.O., Moffatt, M.F., Turner, P., Parkhill, J., Loman, N.J., and Walker, A.W. (2014). Reagent and laboratory contamination can critically impact sequence-based microbiome analyses. *BMC Biol.* 12. doi: 10.1186/s12915-014-0087-z.

Smith, A., Popa, R., Fisk, M., Nielsen, M., Wheat, C.G., Jannasch, H.W., Fisher, A.T., Becker, K., Sievert, S.M., and Flores, G. (2011). In situ enrichment of ocean crust microbes on igneous minerals and glasses using an osmotic flow-through device. *Geochem. Geophys. Geosyst.* 12. doi: 10.1029/2010gc003424.

Smith, D.C., Spivack, A.J., Fisk, M.R., Haveman, S.A., Staudigel, H., and and the Leg 185 Shipboard Scientific Party (2000). Methods for quantifying potential microbial contamination during deep ocean coring. *ODP Tech. Note*, 28. doi:10.2973/odp.tn.28.2000.

Song, Z.Q., Wang, F.P., Zhi, X.Y., Chen, J.Q., Zhou, E.M., Liang, F., Xiao, X., Tang, S.K., Jiang, H.C., Zhang, C.L., Dong, H., and Li, W.J. (2012). Bacterial and archaeal diversities in Yunnan and Tibetan hot springs, China. *Environ. Microbiol.* 15**,** 1160-1175. doi: 10.1111/1462-2920.12025.

Tanner, M.A., Goebel, B.M., Dojka, M.A., and Pace, N.R. (1998). Specific Ribosomal DNA Sequences from Diverse Environmental Settings Correlate with Experimental Contaminants. *Appl. Environ. Microbiol.* 64**,** 3110-3113.

Zhang, X., Feng, X., and Wang, F. (2016). Diversity and metabolic potentials of subsurface crustal microorganisms from the western flank of the Mid-Atlantic Ridge. *Front. Microbiol.* 7**,** 363. doi: 10.3389/fmicb.2016.00363.
